# Toward diagnostically relevant isotope-ratio biomarkers: what does MC-ICP-MS still need for standardized measurements?

**DOI:** 10.1007/s00216-026-06517-y

**Published:** 2026-05-01

**Authors:** Daniel Arias Ramirez, Björn Meermann

**Affiliations:** Federal Institute for Materials Research and Testing (BAM), Division 1.1 - Inorganic Trace Analysis (ITALab), Berlin, Germany

**Keywords:** Stable isotopes, Diagnosis tool, Species-specific isotope information, Clinical and biomedical applications, Lateral-resolved isotope information, Hyphenated techniques/MC-ICP-MS

## Abstract

Stable isotope-ratio analysis by inductively coupled plasma-mass spectrometry (ICP-MS) with a multi-collector (MC-ICP-MS) is increasingly used in life sciences, offering mechanistic insight and potential diagnostic specificity. This review asks the following: What limits biomedical isotope-ratio work, and what steps will make results comparable and fit for biomedical interpretation? Our target audience includes analytical chemists, MC-ICP-MS practitioners, liquid chromatography (LC) and capillary electrophoresis (CE) users, metrology and assurance/quality control specialists, and biomedical researchers adopting isotope-ratio workflows. We map the field from bulk delta (*δ*) values to chemical speciation, species-specific isotope ratios, and spatially resolved readouts. Specific choices in sampling, pre-analytics, separation, sample introduction, and instrument setup are linked to the main sources of bias and to the limits of precision and traceability. Recent progress is synthesized across automated clean-up, LC/ICP-MS and CE/ICP-MS workflows, transient-signal handling, and species-specific isotope dilution with enriched spikes. The review provides practical guidance on baseline correction, peak integration, mass-bias correction, scale realization, and uncertainty budgets. Across studies, the bottlenecks are species instability and interconversion, matrix and space-charge effects, sample-spike mismatch, spectral interferences, lack of species-specific reference materials, and inconsistent operating procedures. We close with an outlook that prioritizes (i) automation and transient-signal processing, (ii) matrix-matched species-specific reference materials, (iii) instrument advances for interference control and coupling efficiency, and (iv) harmonized data-processing standard operating procedures. Together, these steps can enable reproducible multi-site biomedical isotope-ratio workflows and support their translation towards clinical applicability by clarifying where MC-ICP-MS already adds value and where research is still needed.

**Graphical abstract:**

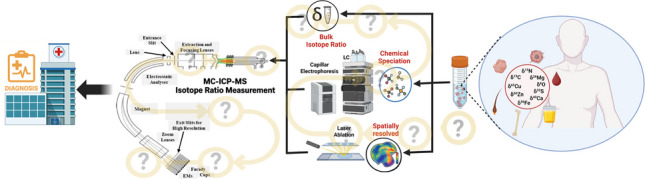

**Supplementary Information:**

The online version contains supplementary material available at 10.1007/s00216-026-06517-y.

## Introduction

Stable isotope analysis has great potential to become a key tool in medical and clinical research because it adds information that elemental concentrations cannot provide. Concentration tells us how much of an element is present. Isotopic composition tells us how that element moves and transforms, and where it comes from. Stable isotope methods track small shifts in the natural abundance of an element’s isotopes. Those shifts reflect biochemical processing, sources, transport pathways, and compartment links within biological systems.

To capture this added information, researchers need isotope-ratio platforms that deliver stable, reproducible ratios in complex biological matrices. Over the last two decades, multi-collector-ICP-MS (MC-ICP-MS) and isotope-ratio mass spectrometry (IRMS) have become robust options for biomedical studies, while thermal ionization MS (TIMS) still supports select applications [1–3].

This progress pushes isotope-ratio measurements towards clinical applicability. PubMed time-series show linear growth in liquid chromatography–mass spectrometry (LC–MS) and gas chromatography–mass spectrometry (GC–MS) publications (1997–2023, LC–MS ≈ 3908 articles/year; 1995–2023, GC–MS ≈ 3042 articles/year), with ~ 6000 LC–MS and ~ 4000 GC–MS records appearing in just the first 7 months of 2024. Inductively coupled plasma-mass spectrometry (ICP-MS) remains less frequent overall, but it fills the metal-ion niche in human biological samples. Together, these trends signal consolidation rather than novelty, and they move isotope-ratio analysis towards a potential biomarker-based diagnostic approach for metabolic, pathological, and therapeutic questions [4].

To interpret isotope ratios in biology, researchers rely on isotope fractionation. Fractionation separates heavy and light isotopes because isotope mass slightly changes bond strength and reaction or transport rates, which shifts isotope ratios. Researchers often distinguish mass-dependent fractionation (MDF) from mass-independent fractionation (MIF), but MDF dominates in most biological settings. Equilibrium fractionation arises because bonds involving the heavier isotope have slightly lower zero-point energy and are therefore marginally more stable; as a result, heavy isotopes preferentially accumulate in the species or compartment with stronger bonding (often associated with a higher oxidation state or a more rigid coordination environment). Kinetic fractionation arises because lighter isotopes react, diffuse, or volatilize faster; unidirectional fluxes and incomplete reactions therefore enrich products in light isotopes and leave the residual pool relatively heavy. In living systems, both mechanisms can operate at the same time, so measured isotope ratios integrate multiple steps across compartments and pathways [4, 5].

In human biology, metabolism drives isotope fractionation through redox cycling (e.g., Fe^2^⁺/Fe^3^⁺, Cu⁺/Cu^2^⁺) [6–8], ligand exchange (metalloprotein binding vs. low-molecular-mass chelates) [9], and compartmental transport (plasma ↔ tissues ↔ excreta) [10]. These isotope-ratio shifts do not come from laboratory artifacts. Biology itself produces isotope discrimination, so isotope ratios can act as intrinsic process-level biomarkers.

Copper provides a clear example. In Wilson’s disease, impaired liver–bile transport and altered ceruloplasmin turnover shift blood ^65^Cu/^63^Cu ratios, and these shifts track hepatic injury and disease progression [11, 12]. In oncology, several studies report that serum copper isotope ratios move toward isotopically lighter values months before classical biomarkers change, consistent with altered intracellular complexation and copper partitioning during metabolism [2]. These isotope ratios therefore report systemic disruption of copper handling, and they can add mechanistic insight beyond concentration data alone.

Other elements show the same diagnostically useful patterns beyond copper. Zinc, sulfur, iron, and calcium isotope ratios each report different parts of metabolism and tissue remodeling in human disease contexts. Zinc supports metalloenzymes and transcription factors, and it shows tissue-specific fractionation. Breast tumors often display isotopically lighter Zn (e.g., ^66^Zn/^64^Zn) than adjacent healthy tissue even when serum stays unchanged, which points to altered metalloprotein partitioning [13–15]. Sulfur is a core component of cysteine, methionine, and disulfide bonds. In blood, ^34^S/^32^S shifts are often small. Yet studies report measurable sulfur isotope differences in liver disease and in some untreated cancers, consistent with altered amino-acid and redox metabolism [13, 15, 16]. Iron isotope ratios (e.g., ^56^Fe/^54^Fe) track redox cycling and redistribution across hemoglobin, storage, and transport pools, and studies link iron isotope shifts to disorders from hereditary iron overload to anemia of chronic disease [13, 15]. Calcium isotope ratios (e.g., ^44^Ca/^42^Ca) reflect bone turnover because lighter Ca isotopes enter hydroxyapatite more readily [13, 16]. Together, these element-specific isotope signatures complement concentration data by encoding pathway activity, and they support mechanism-aware biomarkers for diagnosis, staging, and therapy monitoring [13, 14].

To compare biologically driven isotope shifts across studies, researchers usually report isotope data as relative *δ*-values on internationally defined scales. In *δ*-notation, a sample’s heavy/light isotope ratio is expressed relative to a reference:

$$\delta^nE\left(\%\right)=\left[\left(R\_sample/R\_standard\right)-1\right]\times1000$$

**Fig. 1:**
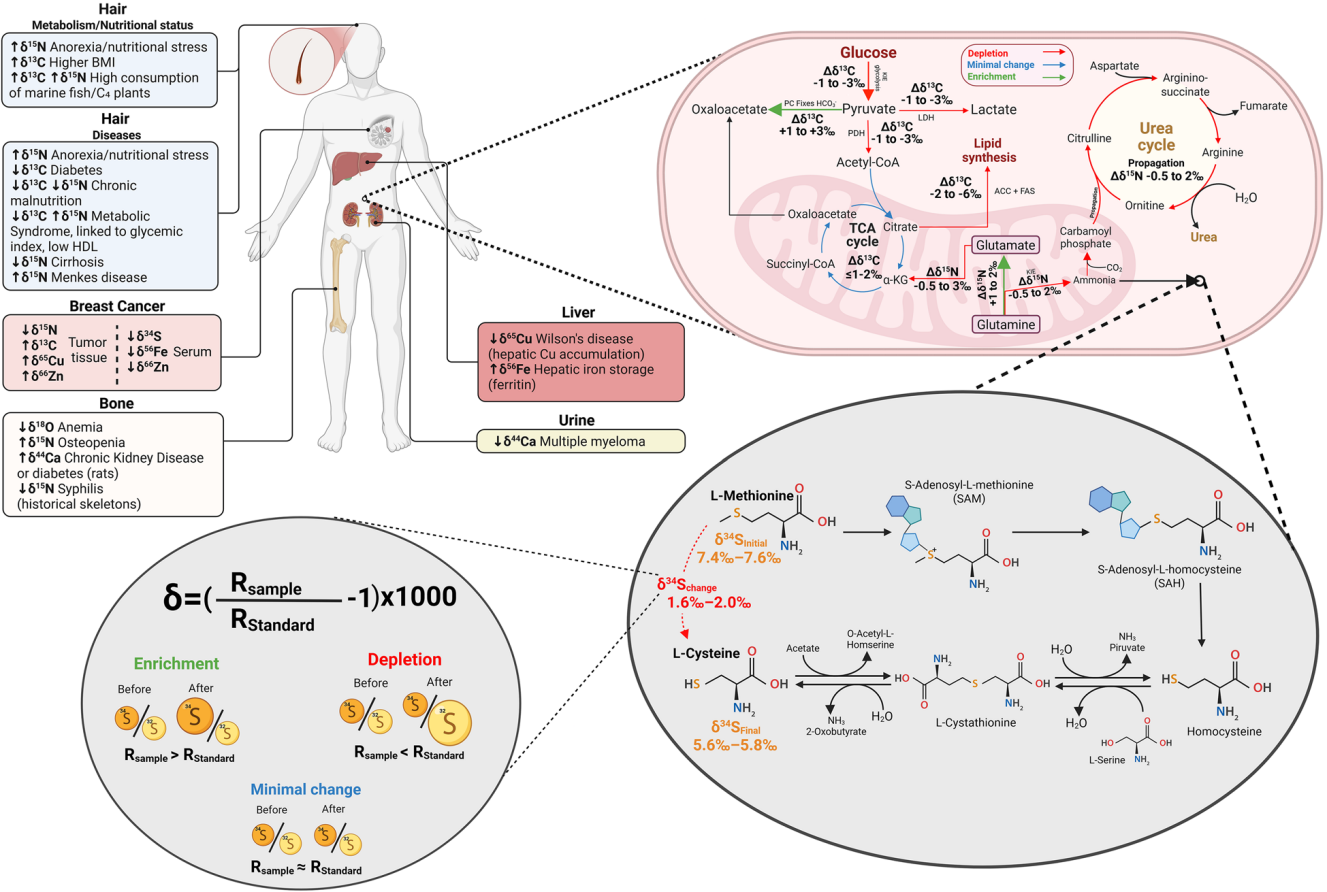
Multi-element δ-mapping in human biology: From fractionation to biomarkers [18]. Figure was created with BioRender (agreement number TM29FG4TLG)

Here, *R* is the heavy/light isotope ratio. Positive *δ* values mean heavy-isotope enrichment relative to the reference, while negative *δ* values mean depletion [17]. Figure 1 summarizes what *δ* means and what “traceable scale” implies in practice.

From a metrology standpoint, the main risk is not *δ*-notation. The main risk is poor or unclear scale realization. Studies become comparable only when they use the same reference scale and when they link local measurements to that scale in a transparent way. The “standard” therefore refers to an internationally recognized reference material. Laboratories often realize the scale through two-point normalization (primary and secondary anchors). Many labs also use in-house working standards for routine runs, so authors should explicitly tie results to the accepted community scale (e.g., VCDT for sulfur; established community anchors for metals; see Table S1, Supplementary Information) [19].

This traceable link is what makes *δ*-values comparable across laboratories. Without clear reference material(s), apparent offsets can reflect different scale realizations instead of true biological differences. Reporting should therefore state the exact ratio measured (e.g., ^34^S/^32^S), the reference scale/material(s), external precision (1σ or 2σ), and the measured fraction (bulk vs. species-specific). If a study needs absolute isotope-amount ratios rather than *δ*-values, it should also state the adopted reference ratio and its reporting convention to avoid hidden scale offsets. In biological matrices, isotope shifts are often small—often < 1 ‰, and sometimes several ‰—so repeatability, instrument stability, matrix handling, and rigorous scale assignment usually dominate the overall uncertainty.

Biomedical samples are complex, so isotope-ratio readouts only help when the workflow keeps the ratio interpretable. Hyphenated inductively coupled plasma-mass spectrometry (ICP-MS) does this by coupling a separation or imaging system to ICP-MS, so the measurement retains chemical identity or spatial context instead of collapsing everything into a single bulk signal. Over the past decades, LC/ICP-MS, capillary electrophoresis/ICP-MS (CE/ICP-MS), and laser ablation/ICP-MS (LA/ICP-MS) have become established platforms for elemental speciation and quantification in medicinal and biomedical analysis [18, 20–24]. Together, these hyphenated approaches enable bulk, species-resolved, and spatial isotope-ratio readouts, which the next paragraph maps to the main instrument platforms.

Hyphenated ICP-MS workflows deliver three complementary readouts: bulk isotope ratios, species-resolved isotope ratios (speciation/SSIR), and spatially resolved isotope ratios. MC-ICP-MS, IRMS, and TIMS anchor bulk ratio metrology when studies prioritize traceability, accuracy, and rigorous uncertainty evaluation. LC/ICP-MS and CE/ICP-MS support species-resolved work because they separate chemical forms under near-physiological conditions, and they can extend to SSIR when isotope-ratio-capable instrumentation is available (often MC-ICP-MS). These workflows must preserve species identity during separation and treat transient signals explicitly, so they can resolve small isotopic variations with confidence [25–28]. For elements that face strong interferences, triple-quadrupole ICP-MS (ICP-QQQ) can add robust interference control through mass-shift strategies, especially for sulfur and selenium [29–31]. When the question is spatial heterogeneity, LA/(MC)-ICP-MS and NanoSIMS provide imaging to map isotope distributions within complex tissues [32, 33]. This platform map sets up the sections that follow by moving stepwise from bulk ratios to species-resolved measurements and, when needed, to spatial mapping for mechanistic interpretation.

**Fig. 2:**
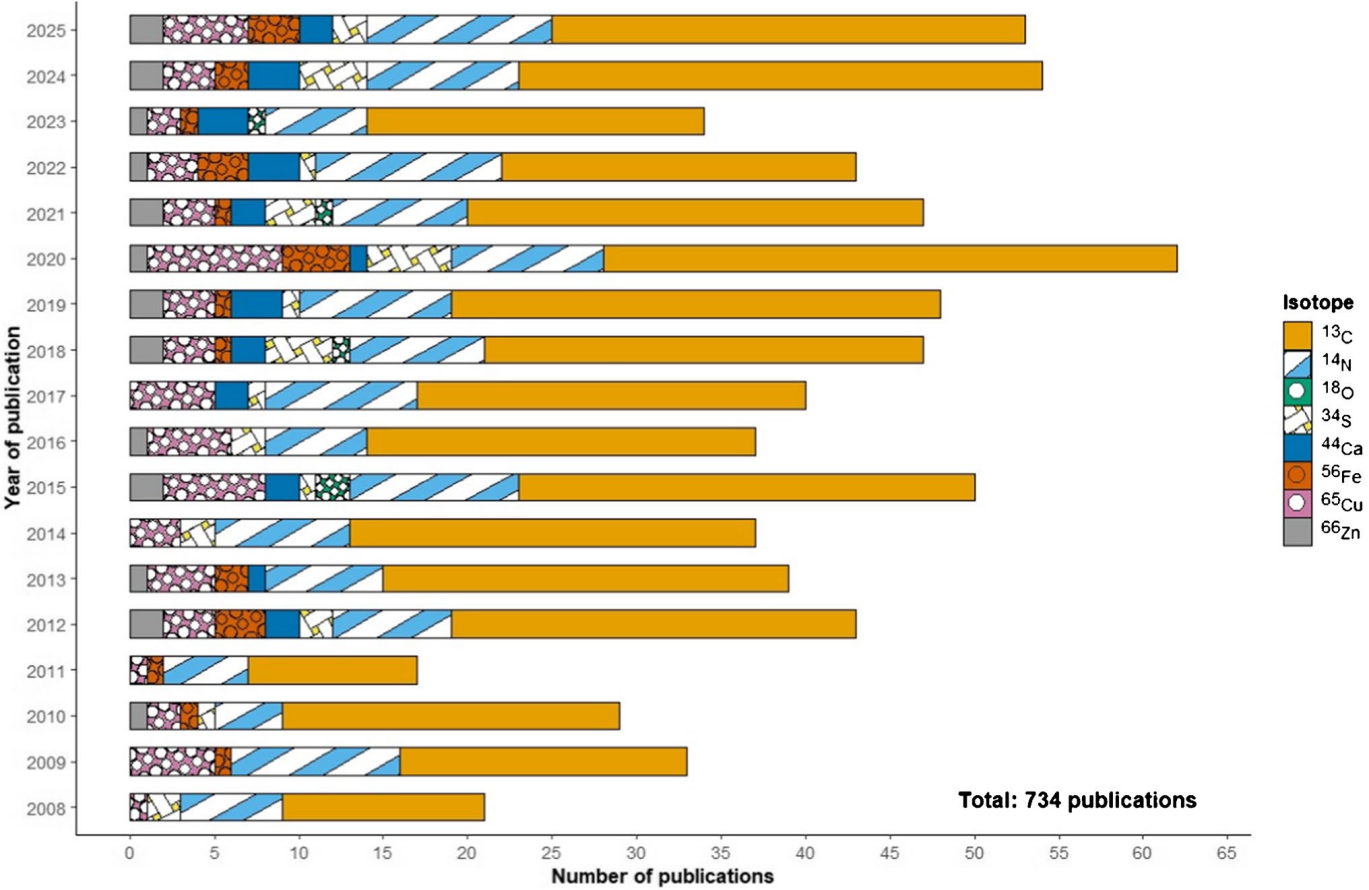
Year-by-year publications on stable isotope applications in a clinical context by element (PubMed 2008–2025; *n* = 734)

Now, to put this in context, how has interest in stable isotope biomarkers grown across isotope systems? We address this question with a bibliometric view of exploratory stable isotope studies in biomedical contexts, where most reports still test biomarker potential rather than routine implementation. PubMed records from 2008 to 2025 (*n* = 734) indicate steady year-on-year growth, with publications spanning the main isotope systems used in this space. Figure 2 corroborates this expansion and shows how the relative contribution of each isotope system shifts over time. For further details, Table S2 (Supporting Information) summarizes studies by isotope system, pathology, and analytical platform.

Stable isotope-ratio analysis has made clear progress toward diagnostic relevance, but most studies remain exploratory. The next step is to move closer to routine healthcare laboratories, where teams can test these methods under realistic constraints and shared standards. This shift will not make isotope ratios diagnostic biomarkers yet, but it will build the evidence base and analytical foundations needed to treat them as credible biomarker candidates. To achieve this, translational studies must consider the entire measurement process as part of the biomarker. That requires tighter control of key analytical factors, including matrix handling and contamination risks, as well as explicit scale anchoring and uncertainty reporting that remain valid across sites and over time. Interlaboratory comparisons on biological reference materials already show that method-related and scale-related offsets can sit in the same per mil range as many biomedical effects, so comparability cannot be optional [34]. Laboratory medicine already relies on metrological traceability and harmonization to make results interchangeable across methods and sites [35]. Stable isotope-ratio work needs the same foundations to operate in practice-ready research across different laboratories.

Building on these foundations, this critical review maps recent developments in stable isotope analysis towards real-world applicability from an analytical chemistry perspective. We address practitioners who use MC-ICP-MS in biomedical settings and teams that translate these workflows into applied biomedical research. The focus is on isotope-ratio measurements that add process information beyond concentration and that remain interpretable in complex biological matrices. The discussion is organized around three focus areas: bulk isotope ratios, species-resolved isotope ratios (speciation/SSIR), and spatially resolved isotope ratios. By tracing how isotopic signatures evolve from whole-sample *δ*-values to species-resolved and spatially contextualized profiles, we show how stable isotope methods can serve as functional biomarkers with mechanistic value. We emphasize how measurement choices govern interpretability and support the move towards real-world applicability. The Technical Challenges section then examines scale realization, calibration architecture, species preservation, and interlaboratory comparability in detail. The Concluding Remarks and Outlook section closes with the authors’ perspective on where the field is headed.

Study selection and inclusion criteria were guided by a structured literature search (cut-off: August 2025). We intentionally exclude radiogenic tracer studies, heavy-label-dosing metabolism trials, purely geochemical/forensic applications, and method papers lacking biological readouts. Given the wide scope of the field, an exhaustive bibliography is beyond the scope of this critical review; any inadvertent omission is unintentional. Readers seeking broader context are referred to authoritative reviews on ICP-MS fundamentals, elemental speciation analysis, isotope fractionation in human disease, and clinical mass spectrometry perspectives.

## Bulk analysis

Bulk analysis provides a screening-level readout by measuring the whole-sample isotopic composition and reporting a single, traceable *δ*-value for the specimen (e.g., *δ*^66^Zn, *δ*^65^Cu, *δ*^56^Fe, *δ*^34^S) referenced to international standards. Because all chemical species of an element are pooled regardless of their molecular form or complexation state, the measured *δ* reflects the total analyte signal. It therefore represents the average isotopic composition across the specimen’s multiple element reservoirs. While bulk analysis cannot separate the contributions of individual chemical forms, it adds a fractionation-sensitive dimension that concentration measurements alone cannot capture. As a whole sample, it enables direct comparisons across cohorts and over time, supports population baselines, and flags system-level shifts with good cost and time efficiency [13]. This traceable *δ*-value then becomes the starting point for later, mechanism-focused stages.

At the instrumental stage that supports bulk *δ* screening and the latter, mechanism-focused steps, MC-ICP-MS remains the gold standard for simultaneous multi-isotope detection [36]. IRMS, especially in continuous-flow mode with gas-source conversion (e.g., to CO₂), provides high-precision *δ*^13^C and *δ*^1^⁸O measurements in clinical and environmental matrices [37]. TIMS offers complementary high-precision isotope-ratio capability [32]. For selected systems (e.g., Sr, Ca, Mg), TIMS can reach ultra-high precision via thermal ionization and static multi-collector arrays, but it requires rigorous chemical purification and careful filament loading, which can constrain throughput in routine clinical workflows. ICP-QQQ (ICP-MS/MS) can help quantify challenging elements, but it typically cannot reach the precision expected for *δ* measurements in clinical settings [38]. Overall, instrument choice defines what is technically feasible, but clinical relevance still depends on validated workflows and metrological controls beyond the platform itself.

Bulk (whole-sample) stable isotope-ratio analysis in biological matrices (serum, plasma, whole blood, urine, tissue) typically follows three steps: (i) acid digestion to remove the organic matrix, (ii) chemical clean-up to isolate the analyte from the matrix, and (iii) dilution/reconstitution to reach target conditions for isotope-ratio measurement by MC-ICP-MS. Chemical purification now acts as a basic requirement for accurate natural-abundance MC-ICP-MS work, and the early idea that MC-ICP-MS could skip extensive clean-up no longer holds (at least in off-line workflows; on-line strategies that can streamline this step are treated later) [39].

Most workflows digest biofluids and tissues with concentrated HNO_3_ plus H_2_O_2_ in closed PFA beakers or closed-vessel microwave systems, then heated on a hot plate (often ~ 110–130 °C, hours to days) to complete oxidation [34, 40]. Published protocols span different sample sizes and reagent volumes, but they follow the same logic: destroy organics first, then convert the residue into the acid medium required for the next separation [34, 40, 41]. Microwave digestion offers a faster closed-vessel route at higher temperature and pressure [42], and some studies still use microwave conditions for whole blood digestion [43]. Dry ashing in a muffle furnace provides an alternative, especially for tissue homogenates [34, 42]. After digestion, labs usually evaporate to dryness and re-dissolve in HCl or HNO_3_ depending on the planned ion exchange chemistry [34]. Sulfur workflows often mirror the same digestion concept and then take up residues in very dilute HNO_3_ before anion exchange purification [44].

After digestion, the key question becomes what remains in solution, because any residual matrix can still distort isotope ratios. Residual matrix can bias isotope ratios through two broad routes: spectral interferences and matrix-driven shifts in instrumental mass bias. Spectral problems can arise from isobaric overlaps, doubly charged ions, and matrix-derived polyatomics, while non-spectral matrix effects can shift the apparent isotope ratio even when no obvious overlap is present. Because matrix correction assumes a specific mass-bias behavior, it can add uncertainty when the interferent’s true fractionation does not follow that assumption [45]. Residual matrix also drives “cryptic” biases via space charge, changes in aerosol transport, and matrix-dependent mass-bias shifts; these effects can reproduce in duplicates yet remain invisible without targeted checks [45]. The practical consequence is that matrix control and verification cannot be treated as optional: acid composition alone can shift Ca isotope ratios substantially [39], residual Ba^2+^ can bias Zn isotope ratios toward falsely heavy values (determination of zinc isotopic composition in biological tissues), and residual Fe and S can defeat Cr corrections and leave a measurable *δ* offset [46]. Isotope-specific interference patterns and mitigation strategies are treated in depth in the Technical Challenges section. Consequently, the present section focuses on the general requirement: residual matrix must be controlled and verified to avoid both spectral and non-spectral bias in *δ* values.

At the clean-up stage, element-specific chemical separation largely governs blanks and spectral interferences. Fe, Cu, and Zn are typically purified on strong anion exchange resins using load/wash/elute schemes for AG-MP1 (anion exchange resin) and related resins [40], with alternative workflows tailored to Fe or to Cu/Zn [34]. Whole-blood Fe purification follows the same logic, using anion exchange with HCl-based loading and elution [43]. By contrast, Ca workflows generally rely on cation exchange (AG50W variants) or extraction chromatography (DGA/TODGA), including single-stage DGA approaches and combined schemes that recover Ca and Mg from one aliquot [47, 48]. For Ca, Sr removal must reach extreme levels to suppress Sr^2+^ interferences [47]. Mg is commonly isolated with AG50W-X8 cation exchange using multi-step elution to achieve clean Mg fractions [41]. Sulfur workflows often use AG1-X8 strong anion exchange, where phosphate control is the key constraint [44], and together, these element-specific choices set the achievable purity and, ultimately, the comparability of isotope-ratio results.

Because element-specific separations set the achievable purity, the clean-up stage must be run under explicit blank and recovery control so that *δ* values remain defensible and comparable. Procedural blanks (reagents + digestion + column chemistry) are processed with each batch to quantify contamination and blank contribution [40], and several studies define element-specific blank targets and resin-blank controls, including low total blanks reported for Ca/Mg and sulfur workflows [40, 44, 48]. Stepwise, blank checks then help localize contamination sources [49], while contamination control is enforced through cleanroom practice, purified acids, ultrapure water, carefully cleaned labware and resins, and material choices that minimize Zn contamination [34, 40, 42]. In parallel, near-quantitative recovery is targeted to reduce on-column isotope fractionation risk and support accurate *δ* values, with high yields reported for Fe/Cu/Zn, Ca, S, and Zn dual-column workflows [44, 47, 50]. Many workflows also validate that chemistry does not introduce artefactual fractionation by comparing processed vs. unprocessed standards and by measuring biological reference materials alongside residual-matrix metrics to confirm ≥ 99% removal of key interferences [40]. Finally, blank and baseline checks during acquisition help to flag memory and scattered-ion backgrounds that can bias isotope ratios if left uncorrected [39]. Together, these controls ensure that the reported *δ*-value reflects the specimen rather than blanks or clean-up artifacts, preserving traceability through the transition into instrumental measurement.

While sample preparation reduces matrix effects, accuracy and comparability still depend on correcting instrumental mass bias (instrumental isotopic fractionation) and accounting for temporal drift after measurement. In MC-ICP-MS, instrumental mass bias mainly arises from mass-dependent, incomplete ion transmission from the plasma through the interface and ion optics. Space-charge effects (Coulomb repulsion) in the extracted ion beam and gas-dynamic/thermal effects across the sampler–skimmer interface drive this behavior [49, 51]. The ion beam leaving the ICP is dense, so ions repel each other as they pass into the low-pressure interface. That repulsion, combined with the steep pressure drop, spreads and deflects the beam in an *m*/*z*-dependent way. Lighter isotopes defocus more and are lost more easily than heavier isotopes, so the collector array receives a heavier-biased ion population. This transport effect creates a reproducible, mass-dependent distortion of measured isotope ratios that must be corrected during data reduction [49, 51].

Sample–standard bracketing (SSB) is the most widely used mass-bias correction approach in practice. In SSB, an isotopic reference standard is measured immediately before and after the sample under matched conditions, and then used to correct the sample ratios for mass bias and drift relative to the two bracket standards [52]. Because mass-dependent transmission attenuates light isotopes more than heavy isotopes, SSB applies a mass-fractionation model to map measured ratios back to true ratios, most commonly the exponential law [51]. The chosen correction law and its parameters must reflect the current instrument state and measurement conditions, so they should be checked against the current transmission behavior, detector calibration, and relevant matrix conditions [51]. It is important to keep in mind that SSB relies on an assumed fractionation law (often the exponential law) and on the bracket standards experiencing the same mass-bias behavior as the sample [53]; otherwise, the corrected ratios can retain a systematic offset.

SSB is one of the most widely used correction approaches, but workflows also apply other strategies that further control mass bias and improve robustness. For Fe isotope ratios, many workflows add Ni and use Ni isotope ratios to derive the mass-bias factor that is then applied to Fe ratios, provided the Ni/Fe ratio and concentration match between sample and standard [54]. For Cu isotope ratios, Ga doping provides the same logic, and it can improve precision and lower blanks in micro-samples such as serum and blood [55]. Double-spike methods take a different route. They add two enriched isotopes of the analyte to each sample, and the inversion solves for mass bias and the true sample ratio (and can also absorb fractionation introduced during chemistry) [56]. In biomedical Zn work, double-spike MC-ICP-MS supports routine *δ*^66^/^64^Zn measurements in certified biological reference materials with external precision of about ± 0.05 to ± 0.10 ‰ (2sd) [57]. Interlaboratory studies on biological reference materials report expanded uncertainties on the same order of magnitude (≈ ± 0.10 ‰ for *δ*^56^Fe and ≈ ± 0.05 ‰ for *δ*^65^Cu and *δ*^66^Zn), which sets a realistic benchmark for “bulk *δ*” performance in real matrices [34].

Vanhaecke et al*.* provide a clear example of how digestion, clean-up, and mass-bias correction work together in practice. They fully digested serum with HNO₃/H₂O₂ and then applied a two-step off-line clean-up. They first used Chelex-100 to remove alkali and alkaline-earth ions, and they then used AG-MP1 anion exchange to separate Cu from the remaining transition metals. They measured ^65^Cu/^63^Cu by MC-ICP-MS and corrected mass bias using Ni-assisted internal normalization together with sample–standard bracketing. This design delivered quantitative Cu recovery and robust *δ*^65^Cu values by limiting residual-matrix contributions and reducing the risk of isobaric and polyatomic bias from major serum ions and the plasma gas Argon (e.g., Na⁺/Mg⁺/Ca^2^⁺) [58].

This same serum workflow also supports proof-of-principle clinical discrimination when *δ* values and concentration are read out together. Aramendía et al*.* targeted Wilson’s disease, where serum Cu is often low, but low serum Cu also occurs in healthy infants, so concentration alone gives overlapping ranges. They measured serum *δ*^65^Cu by MC-ICP-MS after complete HNO₃/H₂O₂ digestion and AG-MP-1 anion exchange isolation with near-quantitative Cu recovery, and they optimized medium-resolution settings and the measurement position to avoid the [^40^Ar^23^Na]⁺ contribution at *m*/*z* 63. They corrected instrumental mass discrimination using admixed Ni together with sample–standard bracketing and reported a typical analytical uncertainty of ± 0.20 ‰ (*k* = 2) from replicate processing of a QC serum. In a bivariate “Cu concentration vs. *δ*^65^Cu” readout, the added isotopic axis separated Wilson’s disease patients from infants even when both groups showed low Cu concentrations, which with concentration data alone could not be achieved [11].

As serum workflows show, *δ*-value plus concentration can be clinically informative; bulk isotope analysis is expanding beyond conventional sampling through microsampling and automation. Dried blood spots (DBS) and volumetric absorptive microsampling (VAMS) enable minimally invasive, microliter-scale collection compatible with routine settings, and validated DBS ICP-MS protocols explicitly manage hematocrit effects and filter-paper blanks to strengthen pre-analytical robustness [59]. VAMS validations—together with a miniaturized ICP-QQQ method—also show agreement with venous whole blood for several trace elements using simple, low-volume preparation that can be scaled up [59]. These formats, however, shift the bottleneck to preparation, making optimized acid extraction and, where feasible, automated handling important for preserving precision and limiting operator-driven variability. In parallel, fully automated chromatographic clean-up on reusable extraction resins (e.g., prepFAST-MC with DGA) can deliver single-stage, quantitative separations with low blanks and throughputs up to ~ 12 samples/day while minimizing carryover and on-column fractionation, as demonstrated for simultaneous Sr, Pb, and Nd separations [60]. For Ca-rich matrices, automated DGA-based protocols further increase tolerable Ca loads while maintaining accurate Sr/Pb ratios, helping to suppress Ca-derived polyatomic effects that would otherwise bias measurements [61]. Together, microsampling and automated clean-up reduce pre-analytical variability and increase throughput, bringing bulk isotope workflows closer to routine implementation.

Despite advances in microsampling and automated clean-up, bulk stable isotope-ratio analysis of biological samples by MC-ICP-MS remains limited in ways that still slow clinical translation (many of these topics are covered in the Technical Challenges section). Because a single whole-sample *δ*-value averages across pools, spatial and molecular specificity is lost. Furthermore, compartments, pathways, and species-level fractionation cannot be resolved, tissue heterogeneity is masked, and source mixing cannot be disentangled from metabolic fractionation without higher-resolution readouts. Moreover, digestion (and sometimes ashing) removes native structure, limiting downstream analyses from the same aliquot [49, 62].

On the practical side, the amount of sample needed can be the bottleneck in analysis, because getting a reliable, representative result may require a lot of material and very thorough mixing [50, 62], while preparation remains slow and non-standardized because digestion plus element-specific resin separation has no universal protocol and can take hours to days per batch [50, 63]. Achieving accurate *δ* values further requires stringent control of contamination and matrix effects—sub-permil precision demands clean infrastructure and strict blank tracking [49], residual matrix can drive spectral and non-spectral bias that is reproducible yet hidden unless explicitly diagnosed [39, 45], and near-quantitative recovery is needed because column fractionation, resin-derived blanks, and memory effects can introduce systematic *δ* bias if chemistry is not tightly controlled [46, 49]. Validation is further constrained by scarce matrix-matched, isotope-certified reference materials and by cases where precision outpaces accuracy because small repeatability coexists with bias from incomplete corrections [49, 50], while cost, expertise, and access remain limiting and biological confounders (diet, age, sex, hormones, time-integration) can blur pathology specificity [24, 62–64]. Addressing these constraints through automation, simplified protocols, dedicated clinical instrumentation, improved reference materials, and coordinated interdisciplinary mechanistic studies remains a central development pathway for this field of application.

## Speciation isotope analysis

Species-specific isotope ratios (SSIR) shift the question from “did *δ* values change?” to “which species caused the *δ* values change?”. SSIR separates, identifies, and quantifies native trace-element carriers under near-physiological conditions. This species-resolved view preserves oxidation and coordination states as far as practical, so the isotope signal links to uptake, transport, and biological activity. Bulk totals and whole-sample *δ* values average across carriers, so they can hide clinically relevant heterogeneity when the species distribution shifts.

**Fig. 3:**
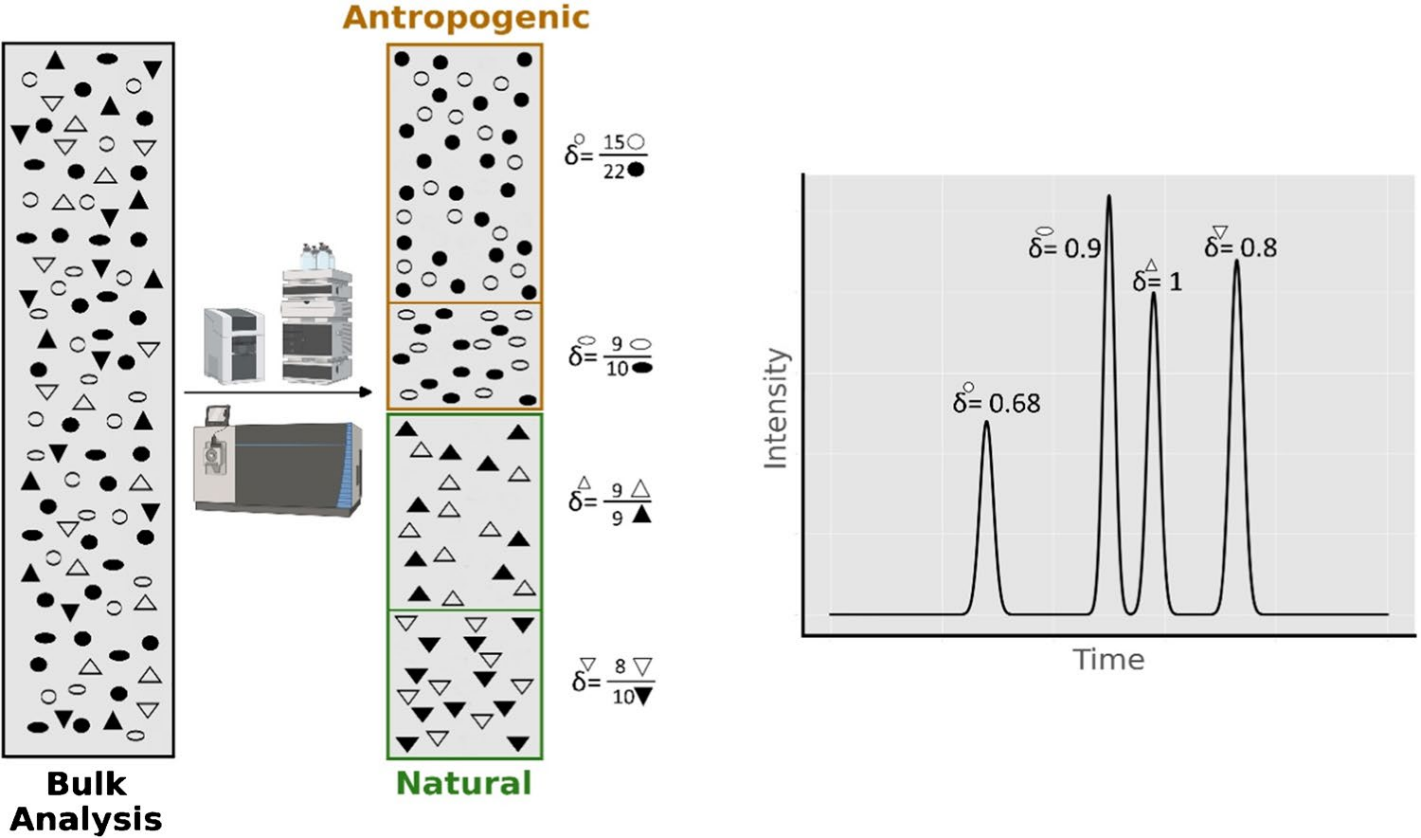
“Peak-resolved *δ* values”—separation/speciation analysis converts a bulk *δ*-value average into species-specific isotope signatures/ratios (SSRI) for source attribution

In practice, common bulk *δ*-value determination neglects elemental species distribution, because the measurement collapses distinct pools into one number (e.g., Cu-ceruloplasmin; Zn-albumin vs. Zn-citrate; Fe in transferrin, ferritin, and heme; discrete Se or Hg species) [25]. Serum *δ*^65^Cu illustrates the point: bulk *δ*^65^Cu can be measured with high precision, yet ceruloplasmin-bound and albumin-bound Cu often exhibit distinct *δ*^65^Cu signatures that track Wilson’s disease, end-stage liver disease, or certain cancers [25, 65]. SSIR resolves which species accounts for the observed *δ*-value shift, so an average change becomes a mechanistic—and potentially actionable—biomarker. This peak/species-resolved perspective also clarifies why bulk analysis can mislead, because the whole-sample signal is a weighted mixture of carrier-specific signatures (*δ*_bulk ≈ *Σ* *fᵢ*·*δᵢ*, Fig. 3).

This species-preserving SSIR approach only works if sample handling and species separation preserve original speciation from sampling through handling and separation. That requirement sets boundary conditions for the sample, its treatment, and the lab environment. Contamination control is central, because even trace inputs can shift oxidation and coordination equilibria before separation. As in bulk analysis, SSIR relies on ultrapure reagents and acid-cleaned labware. However, SSIR extends contamination control beyond elemental blanks: protocols must prevent introduction of complexing agents, oxidizing/reducing agents, or inorganic ions that can shift oxidation states and speciation equilibria—thus, species transformation needs to be avoided [66]. Residual conditioning acids can unintentionally acidify diluted samples and affect species distributions [66]. Strict procedures must ensure that neither tools nor reagents introduce species transformation.

Sample handling is the main safeguard for turning *δ* bulk values into species-resolved *δ* values. To keep carriers intact, SSIR uses aqueous buffers at physiological pH (e.g., TRIS, HEPES, Tricine; pH 7.4–8.6) and isotonic media (e.g*.,* 0.25 M sucrose, or 0.32 M for brain tissue) to keep metalloproteins and low-molecular-mass complexes intact [67]. For biofluids, it minimizes front-end handling—filtration (0.45 or 0.2 µm), dilution, or 10 kDa ultrafiltration—because harsh treatment can redistribute species before separation begins [67], and it applies gentle lysis for blood-derived matrices (freeze–thaw cycles, buffer dilution, high-speed centrifugation) [67]. Recovery alone does not prove that species stayed intact. Gentle water-based leaching can preserve species, but it often extracts only 10–20 %. Stronger chemicals or enzymes can extract much more, but they can also change or break labile species, so the measured species no longer reflect the original distribution [67, 68]. Another option for sample preparation is enzymatic digestion. For example, in the case of selenium, enzymatic digestion can recover most selenium (> 95 %), but it breaks selenium-binding proteins and shifts selenium into other forms/species. This species transformation changes species fractions *fᵢ* (and potentially *δᵢ*), so high recovery does not confirm protein-level speciation or validate the SSIR target [68, 69].

Species transformation and blank level control are the final safeguards to obtain valid information. Keeping samples cold (typically 4 °C during extraction and homogenization; ≤ − 20 °C storage) stabilizes labile equilibria—especially in redox-active systems—and limiting oxygen and light reduces redox- and photoredox-driven species transformation (e.g., As(III)/As(V), Cr(III)/Cr(VI)) [66, 70]. Frozen storage under nitrogen further protects sensitive supernatants [67]. Beyond species transformation, materials and sampling hardware shape species-level losses and contamination: polyethylene or polypropylene vessels reduce adsorption, stainless steel can release Fe, Ni, Cr, and Mn, and hardware can introduce metals that lead to trans-metalation [66, 70].

With species transformation, adsorption, and system blanks controlled, LC/ICP-MS offers complementary, species-preserving separation that resolves element-associated species while maintaining binding equilibria. Size exclusion chromatography/ICP-MS (SEC/ICP-MS) operated in aqueous buffers at near-physiological pH has mapped metalloproteins in paired serum/CSF, and—paired with dynamic reaction-cell ICP-MS technology—can suppress polyatomic interferences while retaining Cu-ceruloplasmin, Zn-albumin (~ 40–80 kDa), transferrin, and ferritin signals [71]. Modern chromatographic columns and low-dead-volume fittings sharpen peaks and reveal redistributions that whole-sample *δ*_bulk cannot resolve [59]. When one dimension is not sufficient for isotopically clean fractions, 2D workflows (e.g., SEC → ion exchange chromatography (IEX)) further improve species purity upstream of the isotope detector and stabilize in-line determination [71].

Charge-based separations provide a second lever to disentangle complexes. IEX/ICP-QQQ-MS discriminates metal–protein/ligand complexes by charge and supports selective ceruloplasmin quantification in Wilson cohorts under volatile ammonium gradients, with reaction-cell chemistry removing overlaps [72–74]. Microflow LC with low-dead-volume interfaces can now be coupled directly to MC-ICP-MS, limiting peak broadening/dilution and enabling real-time, species-specific isotope-ratio measurements [72–74].

For small, polar sulfur species, hydrophilic interaction chromatography (HILIC) or reverse-phase HPLC (RP-HPLC) coupled to ICP-QQQ-MS can apply O₂ mass-shift (SO⁺) to quantify taurine and sulfate with validated transient responses and—using volatile mobile phases—support split-stream to HR-ESI-MS for species identification [75]. Across platforms, mobile-phase design is the main control knob for species integrity: buffered aqueous systems with controlled pH and ionic strength are the default, and organic content is minimized or balanced with volatile additives to protect binding equilibria while keeping the ICP stable [25, 76]. Early pilot studies indicate that carefully optimized low-flow, low-volume on-line interfaces can bring species-specific *δ* measurements closer to off-line performance, but most applications still remain at the proof-of-concept stage [77].

Recent case studies show that on-line strategies can move SSIR from a concept to measurable performance, building on earlier microfluidic, low-dead-volume demonstrations. Tukhmetova et al. reported a species-unspecific on-line isotope-dilution capillary electrophoresis/ICP-MS (CE/ICP-MS) setup in which a post-column ^34^S spike is delivered via a microflow sheath to quantify separated S peaks with minimal dispersion. The workflow achieved baseline separation within 30 min, ng-level S detection, SI traceability to NIST SRM 3154, and serum validation with ~ 7 % uncertainty, illustrating how on-line ID plus low-dead-volume interfacing can underpin accurate, species-resolved S quantification as a basis for isotope-ratio work [78]. In the same direction, CE/MC-ICP-MS delivered direct S-isotope ratios on intact albumin with values comparable to reference measurements, highlighting the benefit of measuring transient peaks without fraction collection [79]. Overall, on-line strategies better preserve species integrity, reduce artifacts associated with extended extraction or fraction collection, and accelerate throughput, whereas off-line routes—still essential for validating purity, recovery, and absolute mass balance—remain more labor-intensive and more prone to species transformation. Collectively, these advances shorten the path from separation to isotopic readout, reduce handling artifacts, and point toward integration of species-specific isotope metrology into routine clinical workflows.

In the same direction, three platform-level innovations are now pushing sensitivity, trueness, and throughput. First, dual- or triple-detector split-stream designs couple ICP-MS with HR-ESI-MS, UV, or fluorescence on synchronized paths, so elemental and molecular signals are acquired in register and complementary confirmation plus chemometric deconvolution reduces misidentification and resolves co-elution [80–82]. Second, two-dimensional and microflow separations (e.g., SEC × IEX, HILIC × RP; microscale LC) further mitigate co-elution while cutting sample consumption and run times—well aligned with minimally invasive formats such as DBS and VAMS [83–85]. Finally, CE offers a complementary separation space at similarly low volumes, but CE/ICP-MS remains technically demanding and low analyte loads can challenge Faraday-cup detection on MC-ICP-MS in terms of sensitivity. Consequently, CE/ESI-MS is often used for molecular confirmation, with CE/ICP-MS largely confined to specialized setups. Overall, these innovations shorten the path from separation to validated species identity and support more reliable, higher-throughput species-resolved readouts.

Despite these platform-level innovations, hyphenated SSIR still faces a shared constraint: separations deliver analytes/species as short, moving peaks rather than a steady aerosol to the plasma, so transient signals are produced by design. This turns isotope measurement into a time-resolved problem, meaning robust SSIR depends on standardized data handling and peak-aware acquisition—not only on separation performance. Automated fraction collection for later MC-ICP-MS, plus chemometrics and machine learning to separate overlapping peaks and split uncertainty sources, can speed up the workflow, reduce user-to-user differences, and improve reproducibility [86–88]. Transient-signal constraints nevertheless remain a practical bottleneck: in hyphenated CE, HPLC/MC-ICP-MS, isotope information is encoded in short, rapidly changing peaks, so limited ion counts and fast intensity changes can amplify noise and bias unless acquisition and evaluation are explicitly adapted to transients [76, 77]. Because interface optimization also shapes the observed peak, minimizing dead volume and matching sheath/makeup flow to nebulizer uptake rates remain essential to prevent dispersion and tailing that erode precision [77, 89].

A concrete illustration of these instrument-limited effects was provided by Gourgiotis et al., who linked transient peak processing to MC-ICP-MS detector physics. They synchronized transient isotope signals by quantifying how quickly each Faraday channel responds via its amplifier time constant, using brief Pb pulses from SRM 981 on Neptune and Neptune Plus instruments to isolate detector-response effects from other fractionation sources [90]. Small response mismatches between amplifier channels manifested as ratio drift across short peaks, i.e., an artifact that grows specifically when signals change rapidly in time. Synchronization based on measured time constants minimized this drift, down to below typical analytical precision when the effective time mismatch fell below ~ 0.001 s [90]. Because these processing choices can measurably shift isotope ratios across short peaks, controlling and reporting them explicitly is one of many steps needed to start moving SSIR towards clinical applicability [90].

A possible way to address this need is IsoCor. IsoCor is an open-source SSIR-data processing software tool (https://apps.bam.de/shn00/IsoCor/)—it supports this by automating baseline correction and peak detection and by benchmarking common transient-signal estimators: peak area integration (PAI), linear regression slope (LRS), and point-by-point (PBP), improving traceability and reducing analyst-dependent choices during QA/QC and method validation [91]. The practical consequences and limitations of these estimators for transient peak SSIR are discussed in detail in the Technical Challenges section. Together, these advances make transient-signal SSIR more comparable across laboratories and accelerate translation to standardized, minimally invasive, high-throughput clinical elemental speciation without sacrificing biological fidelity.

SSIR adds clinical value because it converts a bulk average into species-specific *δ*-value readouts and assigns an observed *δ* shift to the species that changed, which strengthens mechanistic interpretation and supports translation toward standardized, higher-throughput speciation. This gain is only defensible when workflows preserve species integrity and control contamination and transient peak artifacts, because hyphenated SSIR encodes isotope information in short, time-varying peaks that are sensitive to interface and detector dynamics [76–78]. The Technical Challenges section outlines these constraints and other transversal challenges in isotope-ratio analysis that might shape the path towards clinical applicability.

## Technical challenges

To move isotope ratios towards clinical application, the full measurement chain needs to be controlled, not just the instrument. This section maps the transversal technical challenges that still constrain biomedical/clinical isotopic ratio analysis (IRA); solving them will not make isotope ratios diagnostic biomarkers yet, but it will make clinical readouts defensible, and biomarker claims credible. Within this Technical Challenges section, the following parameters are covered: matrix effects and interferences at the *m*/*z* values measured to investigate medically relevant isotopes, coupling and sample introduction, transient signals and data reduction, *δ*-value calculation and mass-bias correction, and finally QA/QC and standardization—CRMs (certified reference materials) and SOPs (standard operating procedures)—plus ring trials, reporting, and uncertainty. Across all platforms, the goal stays the same: making measured isotope ratios track the endogenous composition of clinical analytes, not artifacts introduced by the analytical process.

Clinical translation starts with robust method performance and tight control of sample preparation and matrix effects in complex biological matrices [92]. To turn exploratory screening into a clinically usable bulk *δ*-value test, platform-independent “entry criteria” that make *δ*-values reproducible, traceable, and comparable across time and laboratories are needed. Laboratories must set acceptance targets for within-run repeatability and intermediate imprecision (across days/operators/lots) and demonstrate them using multi-day validation designs. These studies must also verify drift, linearity, and carryover using regular injections of quality control materials and reference materials [17, 93].

Traceability requires explicit isotope-scale realization with at least two anchor reference materials spanning the expected sample range, application of the identical-treatment principle, and full documentation of normalization models and reporting conventions consistent with IUPAC *δ* notation [17, 94–96]. Routine operation must embed fit-for-purpose QA/QC using appropriate controls (matrix-matched where possible), procedural/field blanks, drift monitoring with pre-specified batch-acceptance rules, and interlaboratory exercises (proficiency testing/external quality assessment/ring trials) to quantify and minimize bias [17, 93, 96]. Finally, clinical interpretability requires explicit uncertainty reporting (e.g., expanded uncertainty, *k* = 2) from a documented uncertainty budget that propagates repeatability, reference-material assignments, and the normalization/calibration model, and demonstrates fitness-for-purpose against the intended clinical decision needs [17, 97].

One of the first technical challenges is the inherent complexity of clinical samples: clinical matrices—including whole blood, serum/plasma, urine, and tissue homogenates—challenge isotope-ratio work because proteins, salts, and organic ligands vary across patients and over time [98]. Pre-analytical handling amplifies this variability because collection devices, tube additives, hemolysis, and freeze–thaw cycles can change the chemical background before analysis [99]. Whole blood and tissue homogenates also load the plasma with organic and phosphorus-rich material, which can boost polyatomic formation and worsen suppression effects [100, 101]. Urine adds a different challenge, because ionic strength and pH swing widely, so the same dilution can leave very different residual matrices [75].

This residual matrix biases isotope ratios because it perturbs the physics and chemistry that underpin stable ion transmission and stable mass discrimination. Coexisting salts can suppress or enhance analyte signals and shift extraction and ion-optics conditions, so the same isotopic composition no longer yields the same measured ratio under “matrix-free” tuning [102]. In MC-ICP-MS, matrix-doping studies show that common matrix components can change relative mass-bias behavior and leave systematic errors even after external normalization [103]. Beyond these non-spectral effects, plasma and interface chemistry can also form polyatomic ions from Ar/O and matrix-derived species; even small unresolved contributions can distort natural-abundance isotope ratios, so residual-matrix control remains a precondition for comparable, practice-relevant data.

Spectral interferences are a primary consequence of residual matrix in ICP-MS: the plasma and interface readily form isobaric and polyatomic ions that overlap the *m*/*z* values used to measure medically relevant element isotopes. Typical overlaps include [^64^Ni]^+^⇔[^64^Zn]^+^; [^40^Ar^16^O]⁺⇔[^56^Fe]^+^; [^40^Ar^23^Na]⁺⇔[^63^Cu]^+^; [^16^O^16^O]^+^⇔[^32^S]^+^ and sulfur/oxygen species that impinge on Ca and Fe *m*/*z* windows. If not explicitly managed, such overlaps can dominate the error budget. Mitigation therefore must be layered: rigorous chemical clean-up to strip matrix ions, operation at higher mass resolution where feasible, and targeted reaction/collision cell technology (mass-shift, interference attenuation) in ICP-MS to suppress isobaric interferences [104].

To mitigate these spectral overlaps, interference reduction on MC-ICP-MS often starts by exploiting pseudo-high-mass resolution and integrating interference-free peak shoulders [105]. The resolving power required is interference-pair dependent: modest settings can separate, for example, [^56^Fe]⁺ from [^40^Ar^16^O]⁺ (mass resolution (*R*) about *R* = 3000) [106] or [^32^S]⁺ from [^16^O^16^O]⁺ (commonly in the order of *R* = 2,000) [107]. To achieve the needed *R*, Faraday cups are positioned in a way that only the demonstrably clean part of the ion-beam profile is collected. Typically detection of the respective m/z is conducted on a peak shoulder within the mass spectrum (pseudo-high resolution), rather than at the peak apex where unresolved overlap can persist [108, 109]. This shoulder integration minimizes interference-driven bias without sacrificing precision, and it becomes the first layer before CRC and mass-shift measures when resolution alone is insufficient [109].

Shoulder integration matters most for transient signals (LC/CE peaks), because it restricts the measurement to a demonstrably clean spectral segment and helps damp interference-driven scatter and baseline drift during short elution windows. This narrower integration window can improve precision for short transient chromatographic or electrophoretic peaks without heavy post hoc correction, which is a recurring constraint in hyphenated MC-ICP-MS workflows [110]. The downside is robustness to mass-scale instability: the interference-free shoulder is narrow, so small mass drift can shift the peak relative to a fixed shoulder window and introduce bias [111]. Labs manage this with frequent bracketing or on-line bracketing-like strategies and with run-to-run realignment using the isotope trace. Where available, model-based corrections that track and correct drift—often using amplifier time-constant information and internal signal synchronization—further improve robustness for transient peak work [112].

This shoulder-based strategy generalizes across isotopic systems of biomedical interest. In sulfur isotope analysis for small medical samples, selecting clean peak shoulders has delivered the precision needed to detect diagnostically meaningful biological variations even when sample volumes are extremely limited [44, 113]. For vanadium and calcium, high mass-resolving power separates analyte peaks from a multitude of molecular interferences, producing data quality comparable to TIMS while requiring far less material [114, 115]. Together, these cases underscore the versatility of interference-free shoulder selection across elements and matrices, highlighting its potential as a broadly applicable approach in clinical isotope-ratio analysis [49].

Another instrumental approach to spectral interferences is to operate MC-ICP-MS at higher mass resolution; transmission drops, but tighter bias control usually matters more, and modern ion optics with low-noise amplifiers keep signals usable. Hobin et al*.* illustrate this in practice: on a Neptune XT, “extra-high-resolution” (*R* ≈ 15,000) moves the ^41^K/^39^K measurement onto an [ArH]⁺-free flat shoulder (~ 0.006–0.007 amu), while jet-type cones, a hot plasma, and Aridus-II desolvation compensate for much of the lost transmission [116]. This configuration delivered interference-free *δ*^41^K with long-term external precision of ~ 0.06 ‰ and straightforward SSB to NIST SRM 3141a, and it held across both biological and geological reference materials. The broad, flat shoulder also damped sensitivity to small mass drifts, enabling longer unattended sequences without frequent re-tuning and reducing operator intervention. However, this advantage is not universal: the exact position and width of interference-free shoulders shift with analyte, species, and operating conditions, so labs must measure their instrument response rather than assume a fixed window stays clean. Routine checks with matrix-appropriate CRMs therefore become mandatory to map beam shapes, set defensible integration windows, and maintain traceability and reproducibility across runs and laboratories [49, 113, 117, 118].

A practical way to run shoulder integration at pseudo-high resolution is to use dedicated monitor cups to track flanking masses and adjust windows run to run. Modern collector upgrades—denser Faraday-cup arrays with optimized slits and multiplexed monitoring—let labs collect the analyte on an interference-free shoulder while simultaneously tracking nearby masses for tails, overlaps, and mass drift, so the integration window can be updated in real time or from run to run [114, 119].

Vanhaecke and co-workers demonstrated this approach in clinical microsamples using fixed multi-collector geometries: they positioned the target isotope beams on cups tuned to clean shoulders at pseudo-high-resolution, assigned additional monitor cups to the flanking masses, and realigned the shoulder windows between runs using the monitor channels. This clean-shoulder collection plus simultaneous interferent surveillance stabilized isotope-ratio estimates from tiny cerebrospinal-fluid aliquots while keeping mass-bias correction within a standard bracketing framework, showing how collector design can make shoulder integration practical in real clinical matrices [116, 120, 121].

When shoulders and higher mass resolution still overlap, reaction/collision cell technology is used to remove the respective interferences. The goal stays the same: removing Ar-based and isobaric interferences but keeping simultaneous multicollection so the isotope ratio stays stable. New MS/MS MC-ICP-MS instruments (Thermo Scientific Neoma™ MS/MS and Nu Instruments Sapphire) do this in two steps. First, a mass filter before the cell blocks many unwanted ions (especially matrix ions). Then, the collision/reaction cell (CRC) uses gas to remove what is left. This “filter-then-cell” layout makes the cell chemistry more predictable and reduces side reactions from the matrix. On Neoma, the pre-cell filter reduces the matrix load entering the CRC. The instrument then uses retarding potential quadrupole (RPQ) optics and 10^13^ Ω amplifiers to keep precision high, even for small isotope signals. The CRC can run gases such as O₂, NH₃, H₂, or He, chosen to target specific interferences [122]. On Nu Sapphire, a dual-path architecture (high-energy/non-CRC and low-energy/CRC) allows operation with He–N₂ or He–H₂ to attenuate Ar₂ dimers; recent Se-isotope work reports near-complete Ar₂ suppression and external reproducibility comparable to non-CRC runs while using ~ 40% less Se—useful when sample is limited [123].

Remaining challenges persist even after CRC-based suppression and shoulder integration. Residual tail contributions from strong overlaps can still bias isotope ratios when the integration window is not placed exactly on an interference-free segment. High mass resolution also lengthens acquisition, which lowers throughput and increases exposure to drift and matrix change during a run. These constraints favor adaptive interference control over fixed settings. In practice, peak position and width are tracked in real time to update the integration window, apply model-based tail deconvolution, and run periodic “shoulder-check” or bracketing injections to verify that the chosen window remains interference-free across the sequence. Emerging feedback-driven schemes—including model-based and machine learning approaches—automate these adjustments in response to beam drift and matrix change, reducing operator dependence and interlaboratory variability and moving the field toward standardized, clinic-ready interference control [49, 79, 119, 124].

Coupling often becomes the hidden bottleneck for isotope ratios in biological samples, because the best separation and the cleanest chemistry still fail if the aerosol never reaches the plasma in a stable way. Coupling efficiency—the fraction of introduced sample that becomes ionized and reaches the detector—sets practical detection limits and isotope-ratio precision in ICP-MS and hyphenated formats, and small transfer losses can bias results in trace-level, matrix-rich biomedical samples [125]. Most losses come from hardware choices. Traditional concentric nebulizers often achieve only ~ 1–10 % transport efficiency (TE) because droplets condense or merge in the spray chamber and deposit on walls; serum, blood, and tissue digests can worsen this by promoting aggregation and wall deposition [125]. High-efficiency and microflow nebulizers tighten droplet-size distributions and improve aerosol delivery, which stabilizes signals and can reduce nebulization-linked fractionation across long sequences [58, 125]. Desolvating systems add another lever by stripping solvent vapor, lowering hydride/oxide formation and matrix-driven suppression, and thereby improving sensitivity and reproducibility [126].

Hyphenated LC/ICP-MS and CE/ICP-MS tighten these constraints because the ions arrive as transient signals that are easy to distort (peak broadening, tailing, and fronting). Interfaces must minimize dead volume and use fast-washout spray chambers, so diffusion and dispersion do not broaden or attenuate chromatographic or electrophoretic peaks, since peak shape directly degrades quantification and *δ*-value calculations [126]. CE is especially demanding because flows are typically in the nL min⁻^1^ range, and the electrical circuit must be closed at the outlet. Most designs therefore use a sheath flow to provide enough liquid for stable nebulization (avoiding self-aspiration in concentric nebulizers) and to supply a conductive path to the counter electrode through the sheath liquid [127]. In practice, coupling design is not a detail—it is the gatekeeper for reproducible peak shapes and defensible isotope ratios in biological samples.

Mechanistically, droplet-size distribution sets coupling efficiency by controlling how completely the plasma converts aerosol into ions. Oversized droplets desolvate incompletely and can destabilize the plasma, whereas undersized droplets can evaporate or diffuse away before they reach the interface; an optimized distribution maximizes ion yield [125]. The spray chamber then acts as a selector: its geometry and residence time determine droplet removal, solvent loading, and washout. Heated or desolvating designs and more laminar flow reduce memory effects and carryover and improve recovery and reproducibility across sequences [126].

Sample uptake rate provides a second lever because it balances throughput against solvent load. High uptake promotes solvent loading, raises polyatomic formation, and can destabilize the plasma, whereas ultra-low microflow can improve coupling by limiting aerosol deposition and keeping conditions stable. At the extreme, demountable µ-dDIHEN (direct-injection high-efficiency nebulizer) direct-injection configurations paired with flow-injection valves and gas-displacement pumps can approach near 100 % TE at only a few µL min⁻^1^, sharply improving sensitivity and repeatability [128]. This control over solvent delivery helps keep transient signals comparable from run to run.

Coupling also depends on the fluidics because peak shape and timing are set upstream of the interface. Keep the entire path low dead volume and low pulsation, and design it for fast washout. Stable connections and rapid washout preserve sharp, reproducible peaks in LC/CE or laser ablation (LA) workflows; otherwise, dispersion and delay degrade effective coupling and add systematic error to isotope-ratio calculations [126]. In LA/ICP-MS, efficient extraction/transport of ablated particulates without elemental fractionation is equally determinant, as coupling efficiency links to both spatial resolution and quantitative accuracy in bio-imaging [126, 129].

Optimization is multivariate, so laboratories should tune droplet control, spray-chamber conditions, uptake, and fluidics together with gas flows and RF power instead of changing one factor at a time. Design-of-experiments (DOI) studies can then map robust operating windows for biofluids and tissue digests, and routine tuning with CRMs or internally standardized solutions helps keep coupling performance consistent across runs and instruments [53]. Looking ahead, computational fluid dynamics (CFD) is shaping the next generation of nebulizers and spray chambers by predicting aerosol transport and wall losses. Combined with rapid prototyping and interlaboratory validation, CFD-guided designs can deliver sprays better matched to real clinical matrices and raise coupling efficiency in a more consistent, platform-independent way [125, 126].

To illustrate how interface design can protect both separation and coupling, Taylor et al. compared a non-pneumatic vibrating capillary nebulizer (VCN) with CE-specific pneumatic interfaces (Mira Mist CE; CETAC CEI-100) for on-line CE/ICP-MS. They showed that removing Venturi-driven self-aspiration helps preserve the CE separation and improve effective coupling. VCN delivered 2–4 × lower sensitivity, but it also lowered noise (~ 3.5 × 10^3^ cts·s⁻^1^; RSD ≈ 2.7 %), increased signal-to-noise ratio (SNR), and achieved LOD/LOQ comparable to commercial nebulizers. The design enabled this performance through a low-dead-volume 3D-printed chamber (comprising curtain + carrier gas), a grounded nL min⁻^1^ sheath tee, and Co drift correction—together with a cheap, disposable single-use tip—which minimized carryover and band broadening, supporting reproducible CE peak shapes were obtained [130].

Transient signals make isotope-ratio determination a data assessment as much as an instrument challenge. In LC/CE workflows, peaks are short, so the reported isotope ratio depends on how the time trace is handled. That processing chain includes baseline identification, peak picking, isotope-ratio extraction, mass-bias correction, and standardized *δ*-value calculation. Together, these parameters define the analytical quality of the workflow and expose where the field still needs more robust tools. Data reduction is the bridge from raw transient signals to traceable numbers, and in biomedical studies—where cross-center comparability is critical—small processing choices can shift the final result [49].

Baseline treatment comes first: approaches range from simple pre/post-peak subtraction to moving-median filters, Savitzky–Golay smoothing, SNIP, TopHat, and convex-hull methods [91]. Modern toolkits (e.g., IsoCor) implement these dynamically for transient signals and expose tunable parameters so baseline handling can match matrix and noise characteristics [91]. Because of background drift with introduction conditions and matrix load, choosing and documenting an appropriate baseline strategy is essential to avoid bias that propagates downstream [49, 91].

Peak detection then governs what gets integrated. LC/, CE/, LA/ICP-MS peaks are often only seconds wide; algorithms must identify onsets/offsets as well as the apex under rapidly changing slopes. Beyond point-by-point (PBP) and peak area integration (PAI), regression-based methods such as the linear regression slope (LRS) approach fit rising/falling edges to define boundaries and provide an internal precision estimate from the slope’s standard error [91, 131]. Method choice is matrix-dependent and analyte-dependent, but consistent, optimized algorithms reduce inter-run variability in isotope ratios [91].

With baselines set and peaks delimited, isotope-ratio determination converts intensities to ratios while accounting for drift, cycle-to-cycle fluctuations, and detector noise [49, 131]. For transient signals, misplacing even a small fraction of the window skews isotope ratios; robust practice combines validated integration schemes with replicate strategies appropriate to peak duration [91].

The open-source software IsoCor addresses several of these transient-signal problems by putting baseline handling, peak detection, isotope-ratio extraction, mass-bias correction, and *δ*-value calculation into one automated workflow. It applies the same processing rules to standards and samples, so the result depends less on who clicks the buttons and more on defined parameters. IsoCor also documents settings and exports structured outputs, which support traceability, QA/QC, and cross-center comparison. In practice, this kind of transparent, repeatable data reduction is a practical step that strengthens isotope-ratio workflows towards clinical applicability, because it turns short, noisy peaks into defensible and comparable *δ*-values [91].

Clinical interpretability will also depend on the fit-for-purpose analytical performance defined by clear entry criteria for control and comparability. Under routine conditions, the method must deliver traceable concentrations and defensible isotope information against predefined acceptance targets. That requires documented QA/QC that makes pass/fail decisions explicit and reproducible, not dependent on operator judgment.

Calibration is the main lever for trueness, and two architectures dominate. Species-specific calibration, using authentic standards that match the detected species, maximizes trueness but often cannot be implemented because CRMs and well-characterized spikes are unavailable for many species [132]. Species-unspecific (elemental) calibration with internal standardization is operationally simpler, yet it can bias results when the elemental standard and intact species differ in transport or ionization efficiency. When the unspecific route is unavoidable, accuracy should be protected with matrix-matched standards plus periodic response-factor verification.

In practice, the calibration choices above cannot deliver cross-center trueness if labs lack matrix-matched certified reference materials (CRMs). This bottleneck is most severe for species-specific CRMs and fit-for-purpose enriched spike standards, which remain scarce for many clinically relevant element systems. As a result, interlaboratory comparability weakens and isotopic mass-balance models become harder to validate, because uncertainty in spike purity and incomplete recovery propagate directly into the overall uncertainty budget [25, 133].

To address this materials gap, Schannor et al. developed matrix-matched gelatin “pseudo-CRMs,” bracketed to NIST SRM 976 and verified with secondary reference materials. In LA/MC-ICP-MS, the pseudo-CRMs enabled routine fractionation correction while delivering sub-0.2 ‰ intermediate precision. Crucially, they also resolved clinically relevant *δ*^65^Cu tumor–liver contrasts in situ. This matrix-matched bracketing provides a transferable template when true CRMs or fit-for-purpose enriched spikes are not available [134].

Alongside matrix-matched bracketing, routine clinical control must rely on control charts and predefined acceptance criteria, because clinical decisions require stable day-to-day performance [135]. Start with procedural blanks and carryover tests to separate true signals from contamination and memory [136, 137]. Run replicate preparations and replicate injections (intra-day and inter-day) to quantify short-term and long-term variability. Use bracketing to control drift across sequences. Place control materials on charts to document external reproducibility, and report precision transparently (1σ/2σ and, where appropriate, expanded uncertainty) [138, 139]. Add targeted interference-control checks and isotope-dilution designs [140] off-line or on-line—to strengthen traceability and stabilize transient peak measurements in complex clinical matrices. Together, these elements keep performance comparable across runs and laboratories, which is the minimum requirement for clinical use.

Uncertainty evaluation remains a key gap in biomedical isotope-ratio work. Guideline-conform uncertainty propagation still remains uncommon in biomedical isotope-ratio workflows. Many workflows still report precision but do not carry a guideline-conforming uncertainty through the full measurement model. Laboratories also often miss covariance between corrections, so they can understate uncertainty when several linked steps act on the same data [49, 91, 141]. Without defensible uncertainty, per-mil effects can vanish into noise or look larger than they are, which undermines clinical confidence and cross-laboratory comparability.

Dominant uncertainty sources span the full measurement chain, so no single step can be treated as “the” limiter. Sample preparation adds uncertainty through dilution, digestion, and chemical separation. The instrument contributes mass bias, detector non-linearity, drift, and short-term noise, and calibration plus *δ*-scale anchoring adds further uncertainty through reference-material assignments, normalization choices, and correction models. On top of these analytical terms, matrix effects and biological variability can shift signals and ratios in ways that mimic true isotope effects unless they are modeled explicitly [49, 91, 141]. Interlaboratory uranium isotope ratios in human urine make the consequence concrete: for minor ratios, preparation and separation can dominate the uncertainty budget, so comparable biomedical interpretation requires carrying these terms—along with their covariances—through the full measurement model [142].

Different methods fit different measurement models, so the choice of uncertainty propagation must match the degree of non-linearity and coupling across corrections. GUM-style Gaussian propagation works well when the model is close to linear, and corrections are weakly coupled [143]. Kragten spreadsheet schemes make routine propagation practical and can include covariance terms, but they still struggle once strong non-linearity dominates [144, 145]. Monte Carlo (including adaptive MCM) handles correlated inputs and non-linear correction stacks and returns the full output distribution, which often fits better when many interdependent steps interact across the full measurement chain [146]. This is seen in practice: in triple isotope-dilution MS for total testosterone, adaptive Monte Carlo captured calibration and ratio variability better than closed-form formulas [147], while in ^13^C flux work, EURACHEM-style budgeting helps keep tracer interpretation reliable when natural-abundance signals interfere [148]. Together, these examples illustrate why method choice is not cosmetic: mismatching the propagation approach to the measurement model can understate uncertainty and compromise comparability of biomedical interpretations [149].

Once the propagation approach is chosen, minimum uncertainty reporting must support fit-for-purpose interpretation. The measurand must be defined, and the full measurement model that outputs *δ* values must be stated. Then, the included uncertainty sources must be listed and specified whether biological variability is included or reported separately. The standard uncertainty and the expanded uncertainty with stated coverage and coverage factor must be reported (e.g., *k* = 2) [144] and separated within-run repeatability from intermediate precision when possible. Finally, documentation on how covariance is treated, how the *δ* scale is anchored, and which normalization model is used is mandatory, because these choices can shift *δ* values as much as instrument noise [149, 150].

Persistent gaps remain: Transient signals compress the time available for baseline assignment and integration; peak shapes vary with flow, column efficiency, and instrument state, challenging fixed windows [91, 131]. Heterogeneous laboratory practices—different baseline filters, disparate peak-picking criteria, inconsistent mass-bias models—yield *δ*-values that are not directly comparable across sites [49]. Accordingly, harmonized data-processing SOPs are as critical as instrumental SOPs. Looking ahead, machine learning-assisted pipelines that learn noise structure and peak morphology from large datasets show promise for more resilient baseline subtraction, peak detection, QC flagging, and uncertainty estimation. Keeping these parameters in mind will improve consistency exactly where clinical decisions demand it [91, 141].

## Outlook

Recent advances in ICP-MS are primed to move from promising demonstrations to clinical routine, but doing so will require coordinated progress across the entire workflow—sample handling, metrological traceability, instrumentation, and data analytics.

### First, automation

Traditional isotope-ratio workflows still depend heavily on manual handling, so operator choices can bias results and slow sample processing. Automated platforms address this by combining pressure-driven ion exchange chromatography with robotic sample introduction, which standardizes preparation, reduces human error and contamination (e.g., glove-borne residues), and stabilizes analyte levels. That stability is crucial at trace concentrations and when clinical sample mass is limited [18, 24, 26, 151].

Automation can also extend beyond preparation to species-specific isotope analysis run under near-native, on-line conditions, improving reproducibility while preserving physiologically relevant speciation. By keeping separations on-line, workflows can often avoid harsh digestion and multi-step off-line purification, reducing dependence on costly metal-free cleanroom infrastructure (while still requiring disciplined clean handling). For inter-study comparability, reproducibility must include not only separation and introduction but also how isotope ratios and *δ*-values are computed. The open-source software IsoCor supports this by applying consistent baseline correction, peak detection, mass-bias correction, and *δ*-calculation rules across centers.

### Second, reference materials and spikes

Even strong automation cannot deliver metrological comparability without the right reference materials. Clinical isotope-ratio work needs matrix-matched, species-specific CRMs and enriched isotope spikes to anchor trueness and traceability across labs. As ICP-MS moves toward routine clinical use and multi-site trials, demand for these materials will rise fast and will likely outpace current supply. The field therefore needs coordinated production, certification, and distribution pipelines to avoid bottlenecks that limit validation and comparability [25, 26, 152, 153]. In the meantime, labs are testing simple stand-in materials—such as gelatin-based matrices—that behave more like real clinical samples and can help improve reproducibility and traceability until true CRMs and enriched spikes become widely available [18].

### Third, instrumentation

Biomedical isotope-ratio work still hits hard instrumental limits: spectral interferences, mass fractionation, and memory effects constrain both precision and accuracy, and complex clinical matrices amplify each problem. Labs mitigate these effects with collision/reaction cells, higher mass-resolving power, and stronger chemical separations (often multi-stage ion exchange) to suppress residual overlaps and carryover [27, 117]. These gains still rely on rigorous calibration traceable to robust CRMs, because hardware alone cannot guarantee trueness across runs and laboratories [26].

Instrument design is also evolving. Computational fluid dynamics (CFD) now guides next-generation nebulizer and spray-chamber designs, and teams pair CFD with rapid prototyping and interlaboratory validation to deliver more tailored sprays and higher, more consistent coupling efficiency across platforms [154, 155]. In parallel, feedback-driven control schemes—including model-based and machine learning approaches—can adjust acquisition settings and integration boundaries in real time as the beam drifts or the matrix changes, which reduces operator dependence and interlaboratory variability and moves interference control toward standardized, clinic-ready practice.

A more radical path targets the plasma source itself. Replacing Ar-based ICP with nitrogen MICAP sources on multicollector platforms can remove many argon-derived backgrounds and reduce logistical dependence on Ar. Recent MC-MICAP-MS work shows Sr-isotope precision and accuracy comparable to MC-ICP-MS and, with dry plasma/desolvation, competitive sensitivity, which positions N₂ plasmas as practical, clinic-oriented alternatives [156]. MICAP therefore looks like a promising route for future gains in stable isotope analysis.

### Fourth, data processing and standardization

Reliable, interference-free peak integration in complex transient signals depends on standardization, not ad hoc tuning. Laboratories need consistent calibration routines, stable detector settings, and validated algorithms to keep *δ*-values comparable across runs, operators, and instruments. Data processing should follow a defined workflow—baseline subtraction, peak detection, isotope-ratio determination, mass-bias correction, and standardized *δ*-value calculation—with fixed reporting rules. These decisions set analytical quality and determine whether the readout remains robust enough to support clinically meaningful interpretation.

The field is moving beyond fully manual, deterministic workflows toward reproducible automation. Chemometric and machine learning approaches can automate peak detection, model interference patterns, and harmonize processing across platforms, but only when they are trained, validated, and locked to transparent decision criteria. In parallel, standard bodies and community guidance are converging on shared best practices for data reduction and reporting, which should reduce interlaboratory variability and lower barriers to regulatory adoption [24, 26, 27].

In sum, isotope-ratio work will move towards clinical applicability only if the field advances in lockstep: automation and standardized computation, matrix-matched CRMs and enriched spikes, stronger interference control (CRC and high resolution), CFD-guided sample introduction, adaptive acquisition and integration controls, and consensus data-processing and reporting frameworks. Together, these steps can turn isotope ratios from promising research readouts into reproducible, multi-site biomarker candidates with defensible clinical readouts. There is still a lot of untapped potential.

## Supplementary Information

Below is the link to the electronic supplementary material.

ESM 1 Supplementary Material 1 (DOCX 244 KB)

## Data Availability

Data are available upon request to the corresponding author.

## Declarations

### AI and AI-assisted technologies in the writing process

The manuscript was written without AI tools. All ideas and content originate from the authors and were not AI-generated. AI tools were used exclusively for literature searches but were critically reviewed and checked by the authors for completeness and content. In isolated cases, AI tools (LLM tools) were used: text passages were checked for grammar and spelling, and readability was improved, but was critically reviewed and checked by the authors.
