# Supplementary material for: Toward diagnostically relevant isotope-ratio biomarkers: what does MC-ICP-MS still need for standardized measurements?
Source: Anal Bioanal Chem. 2026 May 1;418(13):3927–48. doi: 10.1007/s00216-026-06517-y (PMC13264568; doi:10.1007/s00216-026-06517-y)
Supplement: Supplementary file 1 — Supplementary Material 1 (DOCX 244 KB) [file 216_2026_6517_MOESM1_ESM.docx]

**Supplementary Information (SI)**

**Toward Diagnostically Relevant Isotope-Ratio Biomarkers: What Does MC-ICP-MS Still Need for Standardized Measurements?**

Daniel Arias Ramirez^1^, Björn Meermann^1*^

^1^Federal Institute for Materials Research and Testing (BAM), Division 1.1 - Inorganic Trace Analysis (ITA_Lab_), Berlin, Germany

**^* Corresponding author:^**

Priv.-Doz. Dr. habil. Björn Meermann, E-Mail: bjoern.meermann@bam.de

Table S1. Commonly reported isotope ratios for selected bio elements and the reference scales/materials typically used for δ-reporting. ^*^

* Compiled from the IUPAC Technical Report on international reference materials for isotope-ratio analysis[1].

| **Element** | **Commonly reported ratio(s)** | **Reference scale / widely used reference material(s)** | **Short metrological note**  **(why it matters)** |
| --- | --- | --- | --- |
| Carbon (C) | ^13^C/^12^C → δ^13^C | VPDB scale; two-point normalization commonly uses NBS 19 and LSVEC | Report VPDB (not “PDB”); explicit scale anchoring avoids inter-lab offsets. |
| Nitrogen (N) | ^15^N/^14^N → δ^15^N | Air-N_2_ (zero-delta scale); common anchors include IAEA nitrogen RMs (e.g., IAEA-N-1) | Scale definition is simple, but routine anchoring is essential for comparability. |
| Oxygen (O) | ^18^O/^16^O → δ^18^O (and ^17^O/^16^O when needed) | VSMOW scale for water; normalization with SLAP; carbonates often reported vs VPDB (with carbonate anchors such as NBS 19) | Always declare scale (VSMOW/VPDB) and normalization approach; mixing conventions creates hidden offsets. |
| Sulfur (S) | ^34^S/^32^S → δ^34^S | VCDT scale; key IAEA sulfur RMs include IAEA-S-1 (scale anchor), plus IAEA-S-2 / IAEA-S-3 | VCDT replaces CDT; specifying the exact anchors used is mandatory for inter-study comparability. |
| Calcium (Ca) | δ^44/40^Ca (sometimes δ^44/42^Ca) | NIST SRM 915a historically widely used | Practical ratio choice can be method-dependent (e.g., ^40Ar interference at m/z 40 in ICP); report ratio definition clearly. |
| Copper (Cu) | ^65^Cu/^63^Cu → δ^65^Cu | NIST SRM 976 (widely used; supply issues noted; alternatives exist) | The chosen Cu RM defines the realized δ-scale; switching RMs without proper linking can shift results. |
| Iron (Fe) | δ^56^/^54^Fe (also δ^57^/^54^Fe in some studies) | IRMM-014 widely used (availability issues noted) | Fe isotope work is sensitive to scale anchoring and consistent reporting of ratios and conventions. |
| Zinc (Zn) | δ^66/64^Zn and δ^68/64^Zn | IRMM-3702 (established anchor; replaces older “JMC Lyon” usage) | Small natural variations demand tight inter-lab harmonization; always state the anchor and ratio. |

Table S2. Stable Isotope Approaches to Elemental Metabolism and Disease

| **Isotope** | **Pathology** | **Platform (bulk / SSIR / LA)** | **Reported u(R)** | **Reference** |
| --- | --- | --- | --- | --- |
| C (δ¹³C in CO₂, ¹³C-UBT) | Helicobacter pylori (adult dyspeptic patients) | Bulk — GC/MS ¹³CO₂/¹²CO₂ (validated *vs.* GC-IRMS) | NR | [2] |
| ¹⁸O KIE (*via* ¹³C/¹²C readout) | None — NTP hydrolysis assay | Bulk — LC/IRMS (internal-competition) | NR | [3] |
| C (δ¹³C in fatty acids) | Earthworm feeding behavior — no disease model | SSIR — GC/C-IRMS | NR | [4] |
| C ([U-¹³C₆]glucose enrichment) | Human plasma glucose metabolism — no disease model | SSIR — LC/IRMS (compound-specific), *vs.* GC/MS & GC/C-IRMS | NR | [5] |
| ¹⁵N (nitrate in urine) | Human — endogenous NO production (no disease model) | SSIR — GC-C-IRMS of urinary nitrate | NR | [6] |
| ²H (DNA deoxyribose from ²H₂O) | CLL — B-cell proliferation in vivo | Bulk — GC/P/IRMS after B-cell isolation, DNA hydrolysis | NR | [7] |
| ¹³C/¹⁵N (Arg→Cit) | Murine endotoxemia (NO production marker) | Bulk — LC/MS (LTQ) with derivatization | NR | [8] |
| C (δ¹³C in urinary estrogens) | Human — exogenous *vs.* endogenous estrogen discrimination | SSIR — GC/C-IRMS (estradiol/estrone δ¹³C) | NR | [9] |
| C (δ¹³C of alanine in RBCs) | Human diet — SSB intake biomarker (no disease model) | SSIR — GC/C-IRMS (alanine) | NR | [10] |
| ¹³C (U-¹³C-glucose → metabolites) | Mitochondrial dysfunction in C. elegans | SSIR — GC/MS isotopologue tracing (MFA) | NR | [11] |
| Fe (⁵⁸Fe tracer; ⁵⁸Fe/⁵⁶Fe, ⁵⁸Fe/⁵⁴Fe) | Healthy males — iron uptake from SBR759 | Bulk — MC-ICP-MS (ferrokinetics) | NR | [12] |
| Fe (δ⁵⁶Fe, δ⁵⁷Fe) | Healthy adult males — erythrocytes vs plasma (no disease model) | Bulk — MC-ICP-MS after Fe purification | NR (δ precision ~0.06–0.15 ‰) | [13] |
| Cu (δ⁶⁵Cu) | Breast & colorectal cancer — human serum | Bulk — MC-ICP-MS | NR (δ precision ~0.04–0.06 ‰) | [14] |
| C (δ¹³C of amino acids in hair) | Forensic/biometric — human hair (provenance/diet), no disease model | SSIR — GC/C-IRMS (compound-specific) | NR | [15] |
| C, N (δ¹³C, δ¹⁵N of BZP/TFMPP) | Forensic — seized tablets (no disease model) | SSIR — GC/C-IRMS (compound-specific) | NR | [16] |
| C (δ¹³C of urinary steroids) | Doping control — detection of endogenous AAS misuse (no disease model) | SSIR — GC/C-IRMS (compound-specific δ¹³C of steroid metabolites) | NR | [17] |
| Cu (δ⁶⁵Cu) | Liver cirrhosis — human serum | Bulk — MC-ICP-MS | NR | [18] |
| C (δ¹³C of urinary steroids) | Doping control — testosterone administration; UGT2B17 deletion polymorphism (no disease model) | SSIR — GC/C-IRMS (compound-specific δ¹³C of steroids) | NR | [19] |
| Cu, Zn, Ca, S (review) | Cancer (general; *e.g.*, breast, colorectal, HCC) | Summary of prior work — mainly Bulk (MC-ICP-MS); some SSIR/LA in tissue | NR | [20] |
| C (δ¹³C of urinary steroid metabolites) — also ²H used with deuterated T | Doping control — genotype-dependent metabolism of exogenous testosterone (UGT2B17 del/del *vs.* ins/ins) | SSIR — GC/C-IRMS (CIR confirmation); plus H-isotope ratios with HRMS | NR | [21] |
| N (δ¹⁵N of amino acids) — esp. threonine | Trophic discrimination / biochemical mechanism (no disease model) | SSIR — GC/C-IRMS (compound-specific AA δ¹⁵N) | NR | [22] |
| Mg (²⁶Mg-enriched tracer; δ²⁶Mg) | Rat femoral implant degradation (no disease model) | Bulk MC-ICP-MS (²⁶Mg/²⁴Mg) + LA (LA/ICP-QMS imaging with IPD) | MC-ICP-MS ≈ 0.02 % (k=2); LA ≈ 0.2 % (k=2) | [23] |
| Fe (δ⁵⁶Fe, δ⁵⁷Fe) | Anemia of chronic kidney disease (CKD) — human serum; differentiation of iron-deficiency (ID) vs EPO-related anemia | Bulk — MC-ICP-MS after Fe purification | NR | [24] |
| C (δ¹³C of urinary steroids) | Doping control — musk administration (no disease model) | SSIR — GC/C-IRMS (compound-specific δ¹³C of steroid metabolites) | NR | [25] |
| C (δ¹³C of fatty acids; bulk lipids) | Authentication of *Ophiocordyceps sinensis* origin — no disease model | SSIR — GC/C-IRMS (fatty acids) + Bulk — EA-IRMS (total lipids) | NR | [26] |
| C (δ¹³C of urinary steroids) — steroid isotopic standards | Doping control (no disease model) | SSIR — GC/C-IRMS (working standards for WADA labs) | NR | [27] |
| ²H (D₂O incorporation into intact lipids) | Pathogen growth during infections — method development (no patient cohort) | SSIR — isotopologue-resolved LC/MS of intact lipids (D₂O-labeling lipidomics) | NR | [28] |
| Cu (δ⁶⁵Cu) — bulk serum vs EXCH+UF fraction | Alcoholic liver cirrhosis — human serum | Bulk — MC-ICP-MS (measured on total serum and ultrafiltrable/exchangeable fraction) | NR | [29] |
| ¹³C (IROA® internal standards; isotopologue ratios) | Murine liver metabolomics — no disease model | SSIR — LC/HRMS (Isotope Ratio Outlier Analysis workflow) | NR | [30] |
| C (¹³C/¹²C of DSPC-palmitic acid in BAL) | Surfactant lung delivery in surfactant-depleted rabbits (RDS model; no patient cohort) | Bulk — GC/IRMS on BAL DSPC-PA | NR | [31] |
| Fe (^58^Fe/^54^Fe *via* ^58^Fe tracer) | Iron-related disorders — patients vs controls after oral ^58^Fe | Bulk — INAA and MC-ICP-MS comparison | NR | [32] |
| C (¹³C from HCO₃⁻ into amino acids) | Porcine lung pathogen *Actinobacillus pleuropneumoniae* — heterotrophic C fixation (no patient cohort) | SSIR — GC/C-IRMS (aspartate, lysine); nanoSIMS used for single-cell confirmation | NR | [33] |
| C (δ¹³C of essential amino acids) in dried blood spots | None — landlocked Arctic char (ecotox; Hg exposure + carbon source fingerprinting) | SSIR — GC/C-IRMS (compound-specific AA δ¹³C) | NR | [34] |
| C (¹³C-labeled OA, LA, DHA) | Maternal obesity — altered materno-fetal PUFA transfer (human, cesarean cohort) | SSIR — GC/C-IRMS (compound-specific enrichment in maternal/cord plasma & placenta) | NR | [35] |
| Mo (δ⁹⁸/⁹⁵Mo) in urine | Healthy adults — exploratory urinalysis (no disease model) | Bulk — MC-ICP-MS after BPHA extraction; double-spike correction | External precision ≤ 0.08 ‰ (2SD) on δ⁹⁸/⁹⁵Mo | [36] |
| ¹³C (U-¹³C-stearic & U-¹³C-oleic acids) | Postmenopausal women — postprandial FA metabolism (no disease model) | SSIR — LC/ToF-MS for enrichment; Bulk IRMS for expired ¹³CO₂ oxidation | NR | [37] |
| Cu, Fe, Zn (δ⁶⁵Cu, δ⁵⁶Fe, δ⁶⁶Zn) | Obesity — bariatric surgery (Roux-en-Y), human serum & whole blood | Bulk — MC-ICP-MS | NR | [38] |
| Cu (δ⁶⁵Cu) | Age-related macular degeneration (AMD) — human serum | Bulk — MC-ICP-MS | NR | [39] |
| ²H (D₂O → myofibrillar alanine) | Healthy resistance-trained men — whey vs control (no disease model) | SSIR — GC-pyrolysis/IRMS of alanine (body water D₂O bolus) | NR | [40] |
| C (δ¹³C of serum amino acids) — esp. alanine | Added sugar/SSB intake biomarker in postmenopausal women (no disease model) | SSIR — GC/C-IRMS (compound-specific δ¹³C of AAs) | NR | [41] |
| Cu (δ⁶⁵Cu) ± Cu/Zn ratio | Papillary thyroid carcinoma (PTC) — human plasma & tumor tissue | Bulk — MC-ICP-MS (δ⁶⁵Cu in plasma & biopsies); (Cu, Zn by TXRF) | NR | [42] |
| ²H (δ²H of high-MW lipids: TGs, GDGTs) | No disease model — method for large, polar lipids | SSIR — HTGC–GC/P-IRMS (high-temperature GC with pyrolysis IRMS) | NR | [43] |
| C (δ¹³C of serum androgens; steroid sulfates) | Doping control — testosterone supplementation in women (no disease model) | SSIR — GC/C-IRMS on serum steroids | NR | [44] |
| C (δ¹³C of breath CH₄) | Immune response / vaccination effects on breath methane (no disease model) | SSIR — GC/C-IRMS on CH₄ (with GC-FID concentrations) | NR | [45] |
| Cu (δ⁶⁵Cu) | Autism spectrum disorder (ASD) — pediatric serum & RBCs | Bulk — MC-ICP-MS (natural-abundance δ⁶⁵Cu) | NR | [46] |
| C (¹³C in breath CO₂/CH₄) | Healthy adults — gut fermentation after ¹³C-labeled wheat bran (no disease model) | SSIR — GC/IRMS for ¹³CO₂/¹³CH₄ (GC for H₂/CH₄) | NR | [47] |
| Cu (δ⁶⁵Cu) | Aging — murine brain (healthy mice, 3–12 months) | Bulk — MC-ICP-MS after Cu purification | NR | [48] |
| C (δ¹³C of free fatty acids from TAGs) | None — method development (short-/medium-chain FAs) | SSIR — GC/C-IRMS (compound-specific δ¹³C) with simultaneous FA profiling | NR | [49] |
| S (³⁴S/³²S in protein) | None — proof-of-concept on albumin | SSIR — CE/MC-ICP-MS (online species-specific S isotope ratios) | NR | [50] |

**Bibliography**

1. Brand WA, Coplen TB, Vogl J, Rosner M, Prohaska T (2014) IUPAC Technical Report Assessment of international reference materials for isotope-ratio analysis (IUPAC Technical Report) 1. https://doi.org/10.1515/pac-2013-1023

2. Jordaan M, Laurens JB (2008) Diagnosis of Helicobacter pylori infection with the 13C-urea breath test by means of GC-MS analysis. J Sep Sci 31:329–335. https://doi.org/10.1002/JSSC.200700385

3. Du X, Ferguson K, Gregory R, Sprang SR (2008) A method to determine 18O kinetic isotope effects in the hydrolysis of nucleotide triphosphates. Anal Biochem 372:213–221. https://doi.org/10.1016/j.ab.2007.09.013

4. Dungait JAJ, Briones MJI, Bol R, Evershed RP (2008) Enhancing the understanding of earthworm feeding behaviour via the use of fatty acid delta13C values determined by gas chromatography-combustion-isotope ratio mass spectrometry. Rapid Commun Mass Spectrom 22:1643–1652. https://doi.org/10.1002/RCM.3455

5. Schierbeek H, Moerdijk-Poortvliet TCW, Van Den Akker CHP, Te Braake FWJ, Boschker HTS, Van Goudoever JB (2009) Analysis of [U-13C6]glucose in human plasma using liquid chromatography/isotope ratio mass spectrometry compared with two other mass spectrometry techniques. Rapid Communications in Mass Spectrometry 23:. https://doi.org/10.1002/rcm.4293

6. Houben E, Hamer HM, Luypaerts A, De Preter V, Evenepoel P, Rutgeerts P, Verbeke K (2010) Quantification of 15N-nitrate in urine with gas chromatography combustion isotope ratio mass spectrometry to estimate endogenous NO production. Anal Chem 82:. https://doi.org/10.1021/ac9019208

7. Hayes GM, Busch R, Voogt J, Siah IM, Gee TA, Hellerstein MK, Chiorazzi N, Rai KR, Murphy EJ (2010) Isolation of malignant B cells from patients with chronic lymphocytic leukemia (CLL) for analysis of cell proliferation: Validation of a simplified method suitable for multi-center clinical studies. Leuk Res 34:. https://doi.org/10.1016/j.leukres.2009.09.032

8. van Eijk HMH, Wijnands KAP, Bessems BAFM, Olde Damink SW, Dejong CHC, Poeze M (2012) High sensitivity measurement of amino acid isotope enrichment using liquid chromatography-mass spectrometry. J Chromatogr B Analyt Technol Biomed Life Sci 905:. https://doi.org/10.1016/j.jchromb.2012.07.036

9. Yang S, Zhang D, Xu Y, Wang X, Liu X, Wang S, Wang J, Wu M, He Z, Zhao J, Yuan H (2013) Discriminating the endogenous and exogenous urinary estrogens in human by isotopic ratio mass spectrometry and its potential clinical value. Steroids 78:. https://doi.org/10.1016/j.steroids.2012.11.013

10. Choy K, Nash SH, Kristal AR, Hopkins S, Boyer BB, O’Brien DM (2013) The carbon isotope ratio of alanine in red blood cells is a new candidate biomarker of sugar-sweetened beverage intake. Journal of Nutrition 143:. https://doi.org/10.3945/jn.112.172999

11. Schrier Vergano S, Rao M, McCormack S, Ostrovsky J, Clarke C, Preston J, Bennett MJ, Yudkoff M, Xiao R, Falk MJ (2014) In vivo metabolic flux profiling with stable isotopes discriminates sites and quantifies effects of mitochondrial dysfunction in C. elegans. Mol Genet Metab 111:. https://doi.org/10.1016/j.ymgme.2013.12.011

12. Gschwind HP, Schmid DG, Von Blanckenburg F, Oelze M, Van Zuilen K, Slade AJ, Stitah S, Kaufmann D, Swart P (2014) Iron uptake and ferrokinetics in healthy male subjects of an iron-based oral phosphate binder (SBR759) labeled with the stable isotope 58Fe. Metallomics 6:. https://doi.org/10.1039/c4mt00126e

13. Von Blanckenburg F, Oelze M, Schmid DG, Van Zuilen K, Gschwind HP, Slade AJ, Stitah S, Kaufmann D, Swart P (2014) An iron stable isotope comparison between human erythrocytes and plasma. Metallomics 6:. https://doi.org/10.1039/c4mt00124a

14. Télouk P, Puisieux A, Fujii T, Balter V, Bondanese VP, Morel AP, Clapisson G, Lamboux A, Albarede F (2015) Copper isotope effect in serum of cancer patients. A pilot study. Metallomics 7:299–308. https://doi.org/10.1039/C4MT00269E

15. Jackson GP, An Y, Konstantynova KI, Rashaid AHB (2015) Biometrics from the carbon isotope ratio analysis of amino acids in human hair. Science and Justice 55:. https://doi.org/10.1016/j.scijus.2014.07.002

16. Beckett NM, Cresswell SL, Grice DI, Carter JF (2015) Isotopic profiling of seized benzylpiperazine and trifluoromethylphenylpiperazine tablets using δ13C and δ15N stable isotopes. Science and Justice 55:. https://doi.org/10.1016/j.scijus.2014.08.003

17. Polet M, Van Eenoo P (2015) GC-C-IRMS in routine doping control practice: 3 years of drug testing data, quality control and evolution of the method. Anal Bioanal Chem 407:. https://doi.org/10.1007/s00216-014-8374-7

18. Costas-Rodríguez M, Anoshkina Y, Lauwens S, Van Vlierberghe H, Delanghe J, Vanhaecke F (2015) Isotopic analysis of Cu in blood serum by multi-collector ICP-mass spectrometry: A new approach for the diagnosis and prognosis of liver cirrhosis? Metallomics 7:. https://doi.org/10.1039/c4mt00319e

19. Strahm E, Mullen JE, Gårevik N, Ericsson M, Schulze JJ, Rane A, Ekström L (2015) Dose-dependent testosterone sensitivity of the steroidal passport and GC-C-IRMS analysis in relation to the UGT2B17 deletion polymorphism. Drug Test Anal 7:. https://doi.org/10.1002/dta.1841

20. Larner F (2016) Can we use high precision metal isotope analysis to improve our understanding of cancer? Anal Bioanal Chem 408:. https://doi.org/10.1007/s00216-015-9201-5

21. Piper T, Schänzer W, Thevis M (2016) Genotype-dependent metabolism of exogenous testosterone – new biomarkers result in prolonged detectability. Drug Test Anal 8:. https://doi.org/10.1002/dta.2095

22. Fuller BT, Petzke KJ (2017) The dietary protein paradox and threonine 15N-depletion: Pyridoxal-5’-phosphate enzyme activity as a mechanism for the δ15N trophic level effect. Rapid Communications in Mass Spectrometry 31:. https://doi.org/10.1002/rcm.7835

23. Draxler J, Martinelli E, Weinberg AM, Zitek A, Irrgeher J, Meischel M, Stanzl-Tschegg SE, Mingler B, Prohaska T (2017) The potential of isotopically enriched magnesium to study bone implant degradation in vivo. Acta Biomater 51:. https://doi.org/10.1016/j.actbio.2017.01.054

24. Anoshkina Y, Costas-Rodríguez M, Speeckaert M, Van Biesen W, Delanghe J, Vanhaecke F (2017) Iron isotopic composition of blood serum in anemia of chronic kidney disease. Metallomics 9:. https://doi.org/10.1039/c7mt00021a

25. Wang J, He Y, Liu X, Yang Z, Yang W (2017) Steroid profile and IRMS analysis of musk administration for doping control. Drug Test Anal 9:. https://doi.org/10.1002/dta.2293

26. Guo LX, Xu XM, Hong YH, Li Y, Wang JH (2017) Stable carbon isotope composition of the lipids in natural ophiocordyceps sinensis from major habitats in China and its substitutes. Molecules 22:. https://doi.org/10.3390/molecules22091567

27. Tobias HJ, Brenna JT (2018) High-volume steroid isotopic standards developed as working standards for gas chromatography–combustion–isotope ratio mass spectrometry. Drug Test Anal 10:. https://doi.org/10.1002/dta.2309

28. Neubauer C, Sessions AL, Booth IR, Bowen BP, Kopf SH, Newman DK, Dalleska NF (2018) Towards measuring growth rates of pathogens during infections by D2O-labeling lipidomics. Rapid Communications in Mass Spectrometry 32:. https://doi.org/10.1002/rcm.8288

29. Lauwens S, Costas-Rodríguez M, Delanghe J, Van Vlierberghe H, Vanhaecke F (2018) Quantification and isotopic analysis of bulk and of exchangeable and ultrafiltrable serum copper in healthy and alcoholic cirrhosis subjects. Talanta 189:. https://doi.org/10.1016/j.talanta.2018.07.011

30. Mendez R, del Carmen Piqueras M, Raskind A, de Jong FA, Beecher C, Bhattacharya SK, Banerjee S (2019) Quantitative metabolomics using isotope residue outlier analysis (IROA®) with internal standards. In: Methods in Molecular Biology

31. Giambelluca S, Ricci F, Simonato M, Vedovelli L, Traldi U, Correani A, Casiraghi C, Storti M, Mersanne A, Cogo P, Salomone F, Carnielli VP (2019) Tracing exogenous surfactant in vivo in rabbits by the natural variation of 13C. Respir Res 20:. https://doi.org/10.1186/s12931-019-1124-9

32. Yagob T, van de Wiel A, Bode P, Demir A, van der Wagt B, Krystek P, Wolterbeek B (2019) Measurement of the enriched stable isotope 58Fe in iron related disorders- comparison of INAA and MC-ICP-MS. Journal of Trace Elements in Medicine and Biology 53:. https://doi.org/10.1016/j.jtemb.2019.02.001

33. Konze SA, Abraham WR, Goethe E, Surges E, Kuypers MMM, Hoeltig D, Meens J, Vogel C, Stiesch M, Valentin-Weigand P, Gerlach GF, Buettner FFR (2019) Link between heterotrophic carbon fixation and virulence in the porcine lung pathogen Actinobacillus pleuropneumoniae. Infect Immun 87:. https://doi.org/10.1128/IAI.00768-18

34. Barst BD, Wooller MJ, O’Brien DM, Santa-Rios A, Basu N, Köck G, Johnson JJ, Muir DCG (2020) Dried Blood Spot Sampling of Landlocked Arctic Char (Salvelinus alpinus) for Estimating Mercury Exposure and Stable Carbon Isotope Fingerprinting of Essential Amino Acids. Environ Toxicol Chem 39:. https://doi.org/10.1002/etc.4686

35. Gázquez A, Prieto-Sánchez MT, Blanco-Carnero JE, Ruíz-Palacios M, Nieto A, van Harskamp D, Oosterink JE, Schierbeek H, van Goudoever JB, Demmelmair H, Koletzko B, Larqué E (2020) Altered materno-fetal transfer of 13C-polyunsaturated fatty acids in obese pregnant women. Clinical Nutrition 39:. https://doi.org/10.1016/j.clnu.2019.04.014

36. Zhang J, Li J, Zhang L, Wang Z bing, Sun S ling, Luo Z yu (2020) Precise determination of the molybdenum isotopic composition of urine by multiple collector inductively coupled plasma mass spectrometry. Rapid Communications in Mass Spectrometry 34:. https://doi.org/10.1002/rcm.8658

37. Rodriguez-Morato J, Galluccio J, Dolnikowski GG, Lichtenstein AH, Matthan NR (2020) Comparison of the Postprandial Metabolic Fate of U-13C Stearic Acid and U-13C Oleic Acid in Postmenopausal Women. Arterioscler Thromb Vasc Biol 40:. https://doi.org/10.1161/ATVBAHA.120.315260

38. Hastuti AAMB, Costas-Rodríguez M, Anoshkina Y, Parnall T, Madura JA, Vanhaecke F (2020) High-precision isotopic analysis of serum and whole blood Cu, Fe and Zn to assess possible homeostasis alterations due to bariatric surgery. Anal Bioanal Chem 412:. https://doi.org/10.1007/s00216-019-02291-2

39. Aranaz M, Costas-Rodríguez M, Lobo L, González-Iglesias H, Vanhaecke F, Pereiro R (2020) Pilot study of homeostatic alterations of mineral elements in serum of patients with age-related macular degeneration via elemental and isotopic analysis using ICP-mass spectrometry. J Pharm Biomed Anal 177:. https://doi.org/10.1016/j.jpba.2019.112857

40. Davies RW, Bass JJ, Carson BP, Norton C, Kozior M, Wilkinson DJ, Brook MS, Atherton PJ, Smith K, Jakeman PM (2020) The effect of whey protein supplementation on myofibrillar protein synthesis and performance recovery in resistance-trained men. Nutrients 12:. https://doi.org/10.3390/nu12030845

41. Yun HY, Tinker LF, Neuhouser ML, Schoeller DA, Mossavar-Rahmani Y, Snetselaar LG, van Horn L V., Eaton CB, Prentice RL, Lampe JW, O’Brien DM (2020) The carbon isotope ratios of serum amino acids in combination with participant characteristics can be used to estimate added sugar intake in a controlled feeding study of US postmenopausal women. Journal of Nutrition 150:. https://doi.org/10.1093/jn/nxaa195

42. Kazi Tani LS, Gourlan AT, Dennouni-Medjati N, Telouk P, Dali-Sahi M, Harek Y, Sun Q, Hackler J, Belhadj M, Schomburg L, Charlet L (2021) Copper Isotopes and Copper to Zinc Ratio as Possible Biomarkers for Thyroid Cancer. Front Med (Lausanne) 8:. https://doi.org/10.3389/fmed.2021.698167

43. Lengger SK, Weber Y, Taylor KWR, Kopf SH, Berstan R, Bull ID, Mayser JP, Leavitt WD, Blewett J, Pearson A, Pancost RD (2021) Determination of the δ2H values of high molecular weight lipids by high-temperature gas chromatography coupled to isotope ratio mass spectrometry. Rapid Communications in Mass Spectrometry 35:. https://doi.org/10.1002/rcm.8983

44. Andersson A, Piper T, Ekström L, Hirschberg AL, Thevis M (2023) Usefulness of serum androgen isotope ratio mass spectrometry (IRMS) to detect testosterone supplementation in women. Drug Test Anal 15:. https://doi.org/10.1002/dta.3428

45. Polag D, Keppler F (2023) Effect of immune responses on breath methane dynamics. J Breath Res 17:. https://doi.org/10.1088/1752-7163/ace9f2

46. Ling W, Zhao G, Wang W, Wang C, Zhang L, Zhang H, Lu D, Ruan S, Zhang A, Liu Q, Jiang J, Jiang G (2023) Metallomic profiling and natural copper isotopic signatures of childhood autism in serum and red blood cells. Chemosphere 330:. https://doi.org/10.1016/j.chemosphere.2023.138700

47. Meiller L, Sauvinet V, Breyton AE, Ranaivo H, Machon C, Mialon A, Meynier A, Bischoff SC, Walter J, Neyrinck AM, Laville M, Delzenne NM, Vinoy S, Nazare JA (2023) Metabolic signature of 13C-labeled wheat bran consumption related to gut fermentation in humans: a pilot study. Eur J Nutr 62:. https://doi.org/10.1007/s00394-023-03161-5

48. Lahoud E, Moynier F, Luu TH, Mahan B, Borgne M Le (2024) Impact of aging on copper isotopic composition in the murine brain. Metallomics 16:. https://doi.org/10.1093/mtomcs/mfae008

49. Mhanna T, Grand M, Schiphorst AM, Le Balch R, Rizk T, Bejjani J, Remaud GS, Tea I (2024) Carbon-13-isotopomics and metabolomics of fatty acids from triacylglycerols: overcoming the limitations of GC-C-IRMS for short- and medium-acyl chains. Anal Bioanal Chem 416:5557–5564. https://doi.org/10.1007/S00216-024-05479-3

50. Tukhmetova D, Langhammer N, Vogl J, Meermann B (2025) Online Isotope Analysis of Sulfur in Proteins via Capillary Electrophoresis Coupled With Multicollector ICP-MS (CE/MC-ICP-MS): A Proof of Concept Study. Electrophoresis 46:290–295. https://doi.org/10.1002/ELPS.202400128
